# Adaptation of an L-Proline Adenylation Domain to Use 4-Propyl-L-Proline in the Evolution of Lincosamide Biosynthesis

**DOI:** 10.1371/journal.pone.0084902

**Published:** 2013-12-27

**Authors:** Stanislav Kadlčík, Tomáš Kučera, Dominika Chalupská, Radek Gažák, Markéta Koběrská, Dana Ulanová, Jan Kopecký, Eva Kutejová, Lucie Najmanová, Jiří Janata

**Affiliations:** 1 Institute of Microbiology, Academy of Sciences of the Czech Republic, Prague, Czech Republic; 2 Department of Biochemistry and Structural Biology, Institute of Molecular Biology, Slovac Academy of Sciences, Bratislava, Slovakia; University of Florida, United States of America

## Abstract

Clinically used lincosamide antibiotic lincomycin incorporates in its structure 4-propyl-L-proline (PPL), an unusual amino acid, while celesticetin, a less efficient related compound, makes use of proteinogenic L-proline. Biochemical characterization, as well as phylogenetic analysis and homology modelling combined with the molecular dynamics simulation were employed for complex comparative analysis of the orthologous protein pair LmbC and CcbC from the biosynthesis of lincomycin and celesticetin, respectively. The analysis proved the compared proteins to be the stand-alone adenylation domains strictly preferring their own natural substrate, PPL or L-proline. The LmbC substrate binding pocket is adapted to accomodate a rare PPL precursor. When compared with L-proline specific ones, several large amino acid residues were replaced by smaller ones opening a channel which allowed the alkyl side chain of PPL to be accommodated. One of the most important differences, that of the residue corresponding to V306 in CcbC changing to G308 in LmbC, was investigated *in vitro* and *in silico*. Moreover, the substrate binding pocket rearrangement also allowed LmbC to effectively adenylate 4-butyl-L-proline and 4-pentyl-L-proline, substrates with even longer alkyl side chains, producing more potent lincosamides. A shift of LmbC substrate specificity appears to be an integral part of biosynthetic pathway adaptation to the PPL acquisition. A set of genes presumably coding for the PPL biosynthesis is present in the lincomycin - but not in the celesticetin cluster; their homologs are found in biosynthetic clusters of some pyrrolobenzodiazepines (PBD) and hormaomycin. Whereas in the PBD and hormaomycin pathways the arising precursors are condensed to another amino acid moiety, the LmbC protein is the first functionally proved part of a unique condensation enzyme connecting PPL to the specialized amino sugar building unit.

## Introduction

**Figure 1:**
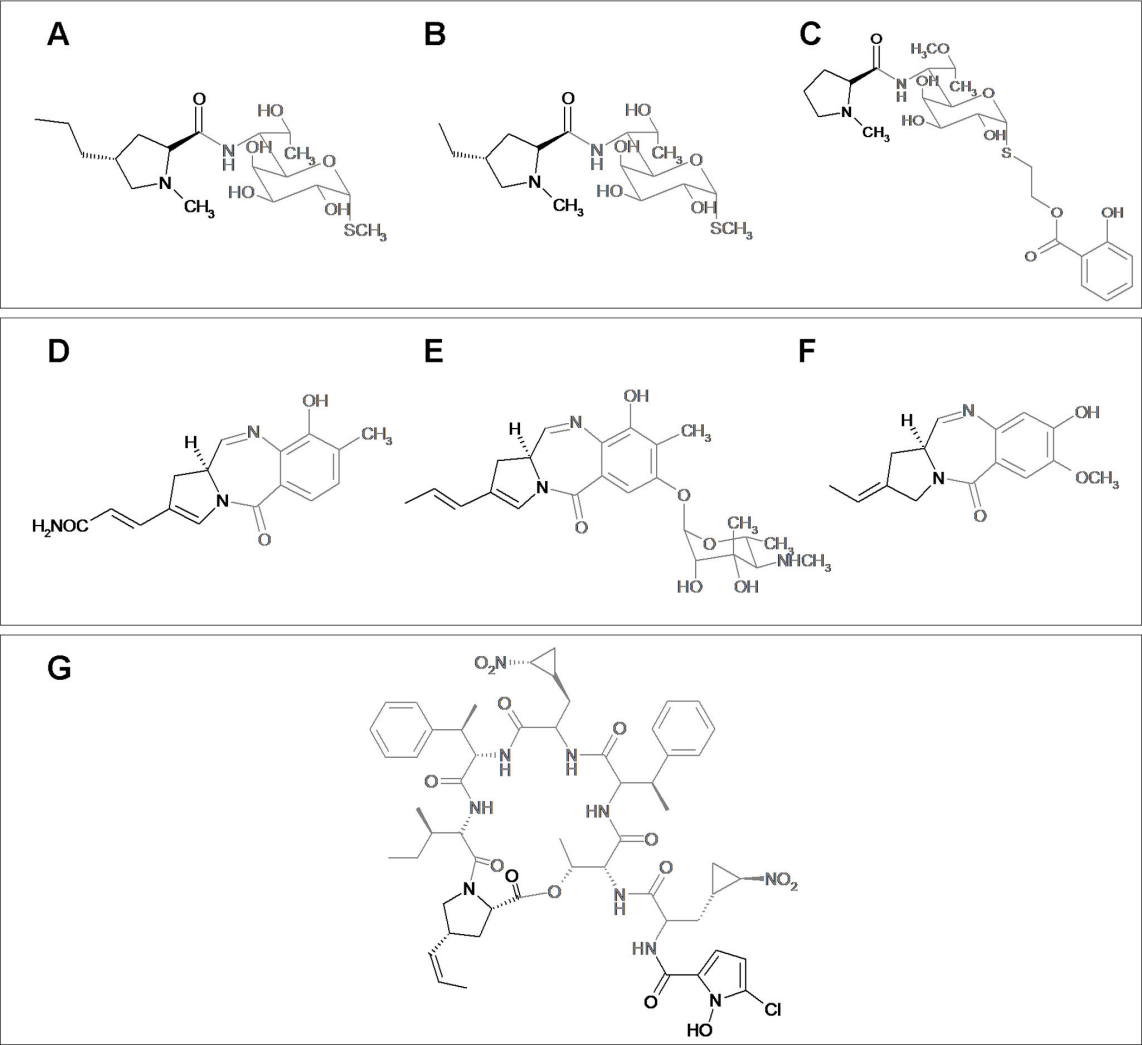
Structures of lincosamides (A–C) and other natural compounds containing branched L-proline precursors (D–G). (A) Lincomycin A, (B) Lincomycin B, (C) Celesticetin, (D) Anthramycin, (E) Sibiromycin, (F) Tomaymycin, (G) Hormaomycin. Fragments derived from L-proline are highlighted.

 Lincomycin and celesticetin are the only natural representatives of the lincosamide antibiotic family (Figure 1A–C). Lincosamides are composed of an amino sugar unit linked to an amino acid via an amide bond. While the biosynthesis of the amino sugar units of lincomycin and celesticetin is quite similar, the biosynthetic origin of the amino acid units profoundly differs. *N*-methyl-L-proline, the amino acid unit of celesticetin, appears to be directly derived from proteinogenic L-proline. The *N*-methyl-4-propyl-L-proline of lincomycin A (lincomycin, unless otherwise specified), on the other hand, arises from the unusual amino acid (2*S*,4*R*)-4-propyl-L-proline (PPL), which is synthetized from L-tyrosine via the oxidative ring opening of L-3,4-dihydroxyphenylalanine (also called L-DOPA) [1-3]. Similar biosynthetic pathways for converting L-tyrosine to rare branched L-proline derivatives with two carbon (2C) or three carbon (3C) side chains are also involved in the biosynthesis of several antitumor pyrrolobenzodiazepines (PBDs; Figure 1D–F) and the *Streptomyces griseoflavus* hormone hormaomycin (Figure 1G), compounds which are structurally and functionally dissimilar to lincomycin. Specifically, six homologs of the lincomycin biosynthetic genes, which presumably encode the proteins responsible for PPL biosynthesis, have been identified in the anthramycin [4] and sibiromycin [5] biosynthetic clusters, but are absent in the related celesticetin biosynthetic cluster. Five of these genes are also present in the biosynthetic cluster of another PBD, tomaymycin [6]. The missing gene in the tomaymycin cluster appears to code for a methyltransferase, and, indeed, tomaymycin’s L-proline derivative has a 2C side chain instead of a 3C one. In lincomycin biosynthesis, an analogous L-proline derivative with a 2C side chain, (2*S*,4*R*)-4-ethyl-L-proline (EPL), is incorporated into a less efficient side product 4´-depropyl-4´-L-ethyllincomycin [7] (lincomycin B; Figure 1B) when the corresponding methylation step in the PPL biosynthetic pathway is omitted. Five homologous genes have also recently been identified in the hormaomycin biosynthetic gene cluster [8]. The presence of shared homologous genes in the biosynthetic clusters of structurally unrelated compounds is an evidence that this set of genes has been spread among the producing strains by horizontal gene transfer (HGT).

The intriguing aspects of the molecular evolution of the lincomycin biosynthetic pathway include not only acceptation of the PPL biosynthetic genes by HGT itself, but also a consequential adaptation step by which *N*-demethyllincosamide synthetase (NDLS), the enzyme which joins the amino acid and amino sugar units, switched from using L-proline to using PPL. A previous study of the lincomycin biosynthetic pathway [9] suggested that NDLS is a multimeric complex, though the individual components were not identified. An obligatory part of NDLS should be an adenylation domain (A-domain) activating the carboxyl functional group of the recognized amino acid. Indeed, an analysis of the lincomycin gene cluster sequence [10,11] (GenBank accession no. EU124663) revealed that *lmbC* gene product shows a sequence homology to A-domains. An orthologous gene called *ccbC* was detected also in the recently sequenced celesticetin biosynthetic cluster from *S. caelestis* ATCC 15084 (GenBank accession no. GQ844764.1). LmbC and CcbC proteins, therefore, seem to recognize and activate the appropriate amino acid (PPL or L-proline precursor, respectively) for the condensation reaction. These two orthologous proteins probably operate as a part of a larger NDLS heteroprotein complex and determine its overall substrate specificity. Although previous attempt to prove the PPL-activating function of the LmbC failed [10], based on considerably increasing number of sequenced and biochemically characterized A-domains, amino acid precursor activation function of LmbC and CcbC can be now firmly assumed.

Several L-proline specific A-domains have been biochemically characterized [12-15], but no A-domain recognizing L-proline derivatives has so far been characterized in terms of kinetic parameters estimation. The objectives of the present work were to demonstrate that LmbC and CcbC function as amino acid activating subunits of the appropriate NDLS, a key enzyme of lincosamide biosynthesis, and, also to compare their substrate specificities and kinetic parameters to elucidate utilization of different amino acid precursors by NDLSs in lincomycin and celesticetin biosyntheses. We also investigated structural aspects of the substrate specificity of these A-domains by using homology models to examine differences in their substrate binding pocket architecture. The results presented here contribute to our understanding of basic principles involved in the molecular evolution of secondary metabolism.

## Results and Discussion

### Protein sequence analysis of CcbC and LmbC

Proteins CcbC (505 amino acid residues; GenBank accession no. GQ912700) and LmbC (509 residues; GenBank accession no. ABX00600.1) share 55.7% sequence identity and contain all 10 core motifs generally conserved in A-domains [16]. A-domains normally form parts of large, multi-domain nonribosomal peptide synthetases (NRPS), but occasionally, individual, stand-alone A-domains are encountered. BLAST search revealed that the closest relatives of CcbC and LmbC are the L-proline specific stand-alone A-domains found in several pyrrole biosynthetic pathways, including coumermycin A_1_, clorobiocin and prodigiosin [17-19].

**Figure 2:**
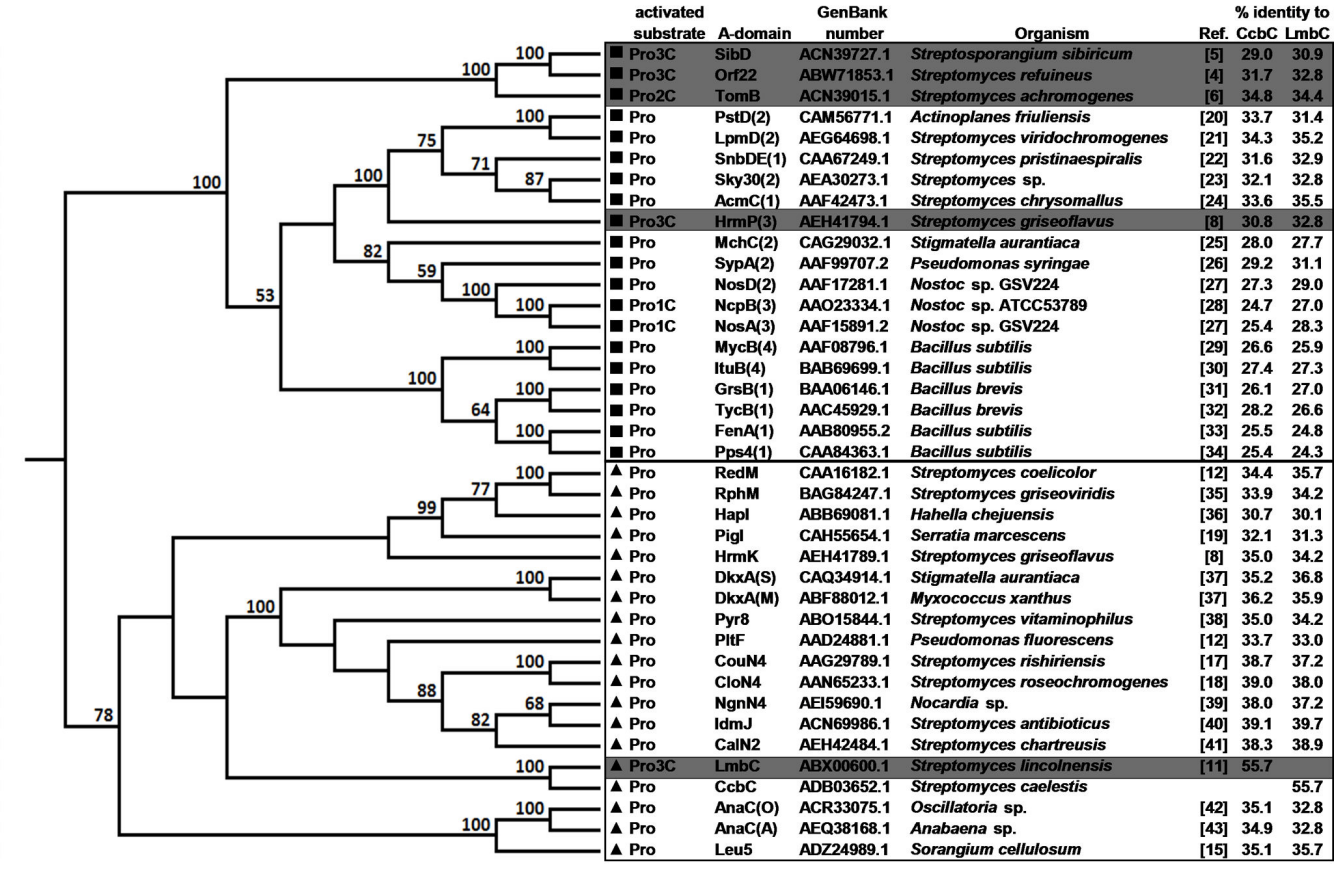
Phylogenetic relationships of NRPS A-domains specific for L-proline or its derivatives. A rooted, neighbor-joining phylogenetic tree was constructed based on the full length amino acid sequences of stand-alone A-domains and excised sequences of modular A-domains. Bootstrap values (100 replicates) above 50% are indicated at the nodes. Modular A-domains are represented by name of respective NRPS module and marked with a ■, stand-alone A-domains are marked with a ▲. The substrates for each domain include L-proline (Pro), L-proline derivatives with one carbon side chains (Pro1C), L-proline derivatives with two carbon side chains (Pro2C), and L-proline derivatives with three carbon side chain (Pro3C). Those A-domains specific for Pro2C or Pro3C substrates are highlighted. Number in parentheses behind the name of respective NRPS denotes the number of the module in NRPS protein chain, if relevant; letter in parentheses denotes the source organism. The GenBank accession numbers, producing strains, and references describing each A-domain are listed. Percent sequence identities with LmbC and CcbC were calculated from pairwise alignments. Figure S1A shows an identical phylogenetic tree reflecting phylogenetic distances, Figure S1B shows the identical set analysis using maximum likelihood method.

A phylogenetic analysis was carried out on a set of sequences of A-domains specific for L-proline or L-proline derivatives. The sequences of all available stand-alone A-domains were used along with a number of representative A-domains with proved or predictable function from multi-domain NRPSs (A-domains which are not stand-alone will hereafter be called “modular” A-domains because they are part of multi-domain NRPS units called modules). Pairwise alignments of each sequence with LmbC and CcbC were carried out to determine the levels of sequence identity. Additionally, a neighbor-joining and a maximum likelihood methods were used to construct phylogenetic trees from a multiple sequence alignment of the entire set. Results including references describing each A-domain [4-6,8,11,12,15,17-43] are summarized in Figure 2 and Figure S1. 

The phylogenetic analysis clearly separated the stand-alone (marked ▲ in Figure 2) and modular A-domains (marked ■ in Figure 2) into two separate clades. Substrate specificity seemed to be a subordinate criterion. Modular A-domains activating L-proline derivatives are split into several separate branches within the clade of L-proline specific modular A-domains. NosA(3)_A_ and NcpB(3)_A_, which adenylate the Pro1C derivative methyl-L-proline (MPL) [27,28,44] evolved from a common ancestor independently of the A-domains activating Pro2C (TomB_A_ from biosynthesis of tomaymycin) and Pro3C derivatives (HrmP(3)_A_, SibD_A_ and Orf22_A_; from biosynthesis of hormaomycin, sibiromycin and anthramycin). The HrmP(3)_A_ belongs, moreover, to a separate branch than the three PBDs biosynthesis A-domains (TomB_A_, SibD_A_ and Orf22_A_).

On the other hand, LmbC, which recognizes a substrate nearly identical to that of SibD_A_, clusters within the clade of stand-alone A-domains, which otherwise adenylate only L-proline. Its closest relative by a large margin is CcbC, with more than 55% sequence identity. None of the other members of this clade has more than 40% sequence identity with either LmbC or CcbC. It seems likely, therefore, that these two proteins arose from a common ancestor. This is supported by the fact that the average sequence identities of CcbC and LmbC with other members of this clade are quite similar (35.6% and 35.1%, respectively). CcbC’s slightly higher similarity reflects the fact that its substrate binding pocket matches that of the other L-proline adenylating A-domains, while that of LmbC has been altered to recognize PPL (see below). Since A-domains which act on branched L-proline derivatives are much less common than those which act on L-proline itself, and since no stand-alone A-domain had been previously identified which acts on these derivatives, it seems logical to assume that the ancestral specificity of both proteins was for L-proline (as in CcbC) and that activation of PPL (LmbC) has developed more recently. Nevertheless we can only speculate, if the ancestor was strictly L-proline specific or exhibited somewhat relaxed substrate specificity. 

### Evolution of nonribosomal code

The A-domain substrate binding pocket is formed by a set of 10 amino acid residues, often called the “nonribosomal code”, whose side chains contact the substrate [45,46]. The substrate specificity of A-domains is determined by a consensus pattern of eight internal amino acid residues of nonribosomal code. Analysis of the nonribosomal code of an uncharacterized A-domain can thus often predict its substrate specificity. Similarly to results of overall sequence homology analysis, also the nonribosomal code analysis distinguishes between stand-alone A-domains and those of modular NRPSs (summarized in detail in Figure S2). L-Proline- or MPL-specific modular A-domains have a VQ(Y/F)IAHVV pattern, which is distinct from that of the stand-alone L-proline A-domains L(L/F)YLALVC. The nonribosomal code of CcbC (V**FY**C**ALVC**), residues identical to the stand-alone consensus are in bold and underlined) logically resembles that of the L-proline specific stand-alone A-domains. On the other hand, the nonribosomal code of LmbC (VALV**A**IG**C**) is rather different, probably as a result of its modification to make use of PPL. Analogically, the radical remodeling of substrate binding pockets of modular A-domains Orf22_A_, SibD_A_, TomB_A_ and HrmP(3)_A_ results in the substrate specificity shift to branched L-proline substrates. However, nonribosomal codes of these A-domains exhibit no similarity to that of LmbC. Thus, the PPL specificity of LmbC evidently evolved independently of that of the modular A-domains.

### Biochemical characterization of CcbC and LmbC

Except for partially characterized HrmP(3)_A_, substrate specificities of all other modular A-domains activating branched L-proline precursors were predicted only based on their sequences and on the formulas of their respective products. The absence of experimentally characterized branched proline derivative specific modular A-domains, together with the lack of their close relatives which specifically adenylate L-proline, hamper the substrate specificity evolution analysis. On the other hand CcbC and LmbC form an orthologous pair of closely related A-domains with different substrate specificities, making them an ideal system to study the types of changes which occur during the evolution of substrate specificity.

**Figure 3:**
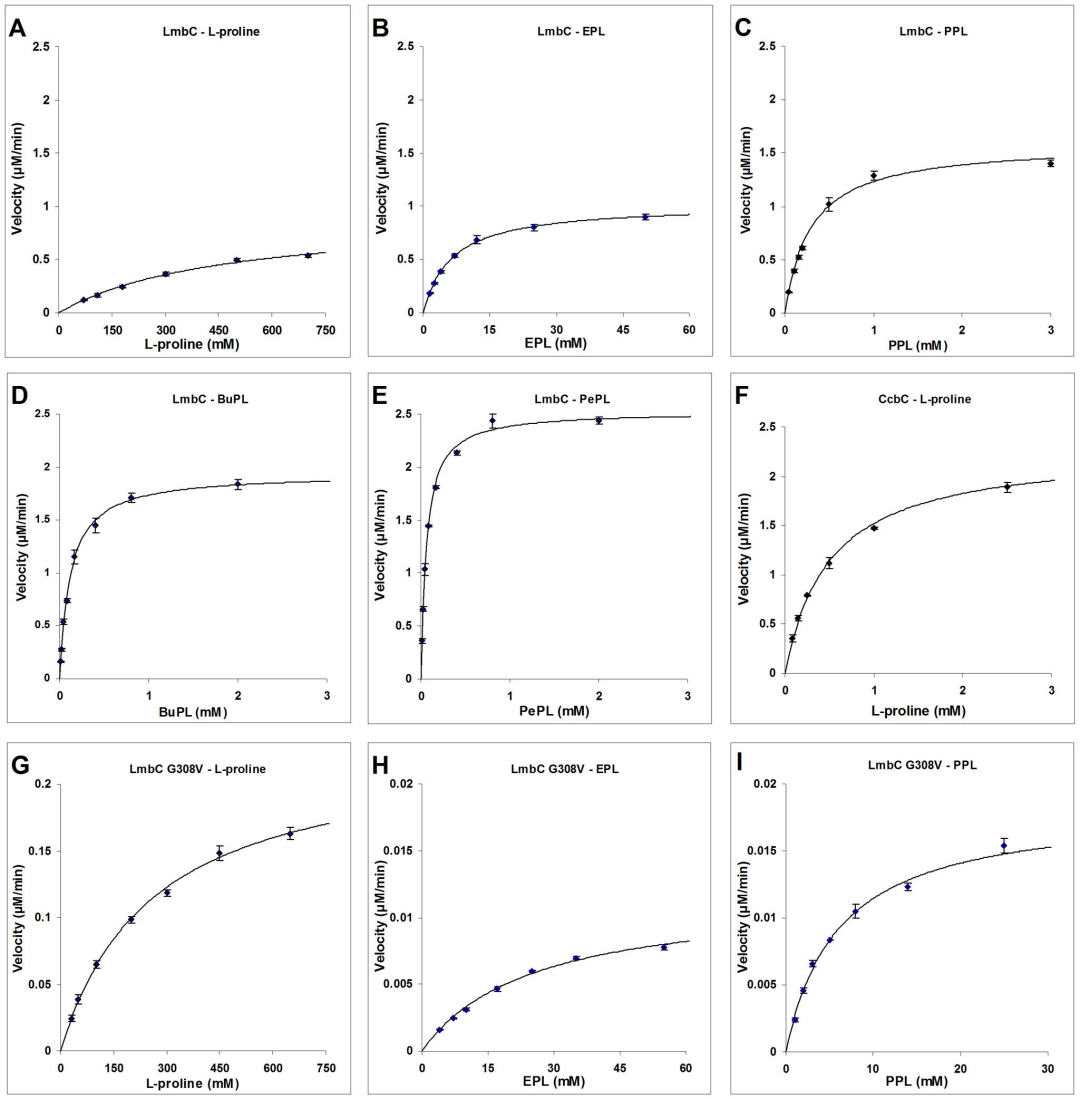
Comparison of the CcbC, LmbC and LmbC G308V reaction kinetics for various substrates. The following combinations of proteins and substrates were tested: (A) LmbC vs. L-proline, (B) LmbC vs. EPL, (C) LmbC vs. PPL, (D) LmbC vs. BuPL, (E) LmbC vs. PePL, (F) CcbC vs. L-proline, (G) LmbC G308V vs. L-proline, (H) LmbC G308V vs. EPL and (I) LmbC G308V vs. PPL. All reactions were performed in triplicate. The error bars indicate the standard deviation. The reaction velocity is expressed as the amount of radioactive ATP (μM) produced per minute at protein concentration 0.05 μM. Reaction conditions are described in Experimental Section.

Soluble, recombinant CcbC carrying an N-terminal His_6_ tag and LmbC carrying a C-terminal His_8_ tag were produced in *Escherichia coli*. Unless otherwise stated, CcbC and LmbC in the following text concerning biochemical experiments, define His-tagged forms of proteins mentioned above. These proteins were stable at –20°C for a few weeks. They were purified in a one-step procedure by nickel affinity chromatography to near homogeneity (Figure S3). Typical yields were 9 mg of pure CcbC per 100 mL of cell culture and 3 mg of LmbC per 100 mL of cell culture. The activities of CcbC and LmbC were determined using an ATP-[^32^P]PPi exchange assay, which measures the transfer of radioactivity from ^32^P-labeled PPi to ATP. The reactions required 4 mM MgCl_2_ and 1 mM ATP. ATP concentrations higher than 1.5 mM rapidly inhibited the reaction. The optimal pH for both proteins was estimated to be 8.7. The kinetic parameters of CcbC and LmbC were assayed with L-proline and its branched derivatives differing in the length of the side chain. The results are shown in Figure 3A–F and summarized in Table 1 and Table S1). 4-Hydroxy-L-proline, L-alanine, L-valine and L-tyrosine were also tested.

**Table 1:** The activity of CcbC, LmbC and LmbC G308V for variable substrates.

**Protein**	**Substrate**[a]	***K*_m_ (mM**)	***k*_cat_ (min^–1^**)	***k*_cat_/*K*_m_ (mM^–1^min^–1^**)
**CcbC**	**L-proline**	**0.5 ± 0.03**	**45 ± 0.9**	**91.7**
CcbC	EPL	ND	ND	ND
CcbC	PPL	ND	ND	ND
LmbC	L-proline	470 ± 60	20 ± 1	0.043
LmbC	EPL	6.4 ± 0.3	22.1 ± 0.3	3.46
**LmbC**	**PPL**	**0.29 ± 0.03**	**34.8 ± 1**	**121**
LmbC	BuPL	0.118 ± 0.008	42 ± 0.8	359
LmbC	PePL	0.0596 ± 0.003	55.2 ± 0.8	925.7
LmbC G308V	L-proline	260 ± 20	5 ± 0.2	0.019
LmbC G308V	EPL	25 ± 2	0.26 ± 0.01	0.01
LmbC G308V	PPL	6 ± 0.7	0.41 ± 0.02	0.068

[a] EPL - (2*S*,4*R*)-4-ethyl-L-proline; PPL - (2*S*,4*R*)-4-propyl-L-proline; BuPL - (2*S*,4*R*)-4-butyl-L-proline and PePL - (2*S*,4*R*)-4-pentyl-L-proline.

Reaction conditions are described in Experimental Section. Rows showing results for LmbC and CcbC with their natural substrates are highlighted. ND – tested, not detectable. The error values indicate the standard error.

CcbC activates its natural substrate L-proline with kinetic parameters (*K*_m_ 0.5 ± 0.03 mM; *k*_cat_ 45 ± 0.9 min^–1^; Figure 3F) similar to those of other L-proline specific stand-alone A-domains (examples given below) and exhibits a strict substrate specificity, having no activity in the presence of PPL or (2*S*,4*R*)-4-ethyl-L-proline (EPL); it will still act on 4-hydroxy-L-proline, but with a *K*_m_ value 23 times higher than for L-proline, though its *k*_cat_ value remains comparable. CcbC thus cannot utilize either of the branched L-proline precursors from the lincomycin biosynthetic pathway. No activity could be detected even in the presence of 50 mM EPL or PPL and at 100 times higher concentration of CcbC than was used in the L-proline assay.

LmbC possesses a broader substrate specificity than CcbC; its kinetic parameters for its natural substrate, PPL (*K*_m_ 0.29 ± 0.03 mM; *k*_cat_ 34.8 ± 1 min^–1^; Figure 3C), are comparable to those of CcbC with L-proline and are within the range of values previously published for the L-proline specific stand-alone A-domains CloN4 (*K*_m_ 0.53 mM; *k*_cat_ 13.1 min^–1^), CouN4 (*K*_m_ 1.16 mM; *k*_cat_ 2.5 min^–1^), RedM (*K*_m_ 1.54 mM; *k*_cat_ 170.9 min^–1^), PltF (*K*_m_ 0.51 mM; *k*_cat_ 332.6 min^–1^), AnaC (*K*_m_ 0.97 mM; *k*_cat_ 68.5 min^–1^) and Leu5 (*K*_m_ 0.017 mM; *k*_cat_ 174 min^–1^) [12-15]. The affinity of LmbC is substantially lower for EPL, an alternative natural LmbC substrate, precursor of lincomycin B (Figure 3B). Thus, the substrate which yields a biologically more efficient product is preferentially activated.

LmbC is able to activate both L-proline (Figure 3A; Table 1) and 4-hydroxy-L-proline, but its affinities for these substrates are ~10^3^ times lower than for PPL. Proteinogenic L-proline is thus effectively excluded from incorporation into lincomycin. Similarly, both CcbC and LmbC do not act efficiently on other, inappropriate proteinogenic amino acids. For example, both proteins activate L-alanine and L-valine, but with ~10^3^ times lower efficiency than their natural substrates. Neither seems to act at all on L-tyrosine, the precursor of PPL.

On the other hand, LmbC is also able to activate not only its natural substrates, but also synthetic L-proline derivatives with longer alkyl side-chains, including (2*S*,4*R*)-4-butyl-L-proline (BuPL; Figure 3D) and (2*S*,4*R*)-4-pentyl-L-proline (PePL; Figure 3E). The kinetic parameters for both these substrates are even better than for PPL. The evolutionary adaptation therefore produced an enzyme with relaxed substrate specificity. This is a frequent phenomenon among secondary metabolism enzymes. The shift from the strict L-proline substrate specificity to the relaxed one for more hydrophobic branched L-proline derivatives of LmbC is also consistent with previous reports that those A-domains which activate hydrophobic amino acids are generally less selective than those which activate polar amino acids [47,48].

The relaxed substrate specificity of LmbC, and thus of N-demethyllincomycin synthetase, along with the relaxed substrate specificity of the *N*-methyltransferase LmbJ which catalyzes the following and final step of lincomycin biosynthesis [49] have important practical consequences. Lincomycin derivatives with extended side-chains on the proline building unit exhibit higher antibacterial and antiplasmodial activities [50]. We have previously made use of the relaxed substrate specificities of LmbC and LmbJ to produce 4′-butyl-4′-depropyllincomycin and 4′-depropyl-4′-pentyllincomycin mutasynthetically in a *Streptomyces lincolnensis* ATCC 25466 mutant strain blocked in PPL production and fed by either BuPL or PePL, as appropriate [51]. Taken together, these results suggest that if the CcbC and LmbC ancestor was specific for L-proline, as argued above, then the differences between the substrate binding pockets of CcbC and LmbC should reflect the changes which needed to take place in order to transform the ancestral protein into something able to act on PPL, a new and structurally modified substrate. In parallel, these changes must also have acted to reduce the affinity of LmbC for its original L-proline substrate which is generally available in the cell pool of proteinogenic amino acids. Logically, radical remodeling of A-domain substrate binding pocket should have been a crucial prerequisite for the NDLS substrate specificity adaptation. It should be noted, that the *k*_cat_ of A-domains in the adenylation reaction may be different from those of the same substrate in the condensation reaction [52]. In the situation when the other NDLS subunits are not identified, the ATP-[^32^P]PPi exchange assay characterizing the A-domain remains the most frequent method to use. This standard method was previously used for the characterization of several stand-alone L-proline specific A-domains [12-15].

In order to understand the differences in substrate specificity between CcbC and LmbC in terms of protein-substrate molecular interactions, and, by extension, between the ancestral protein and LmbC, homology models of both proteins were constructed and structurally validated (i.e. verified to be models of native-like proteins) by molecular dynamics (MD) simulation. Moreover, an LmbC with point mutation G308V in critical position of substrate binding pocket was designed and tested both *in silico* and *in vitro*.

### Homology model construction and structure verification by MD simulation

**Figure 4:**
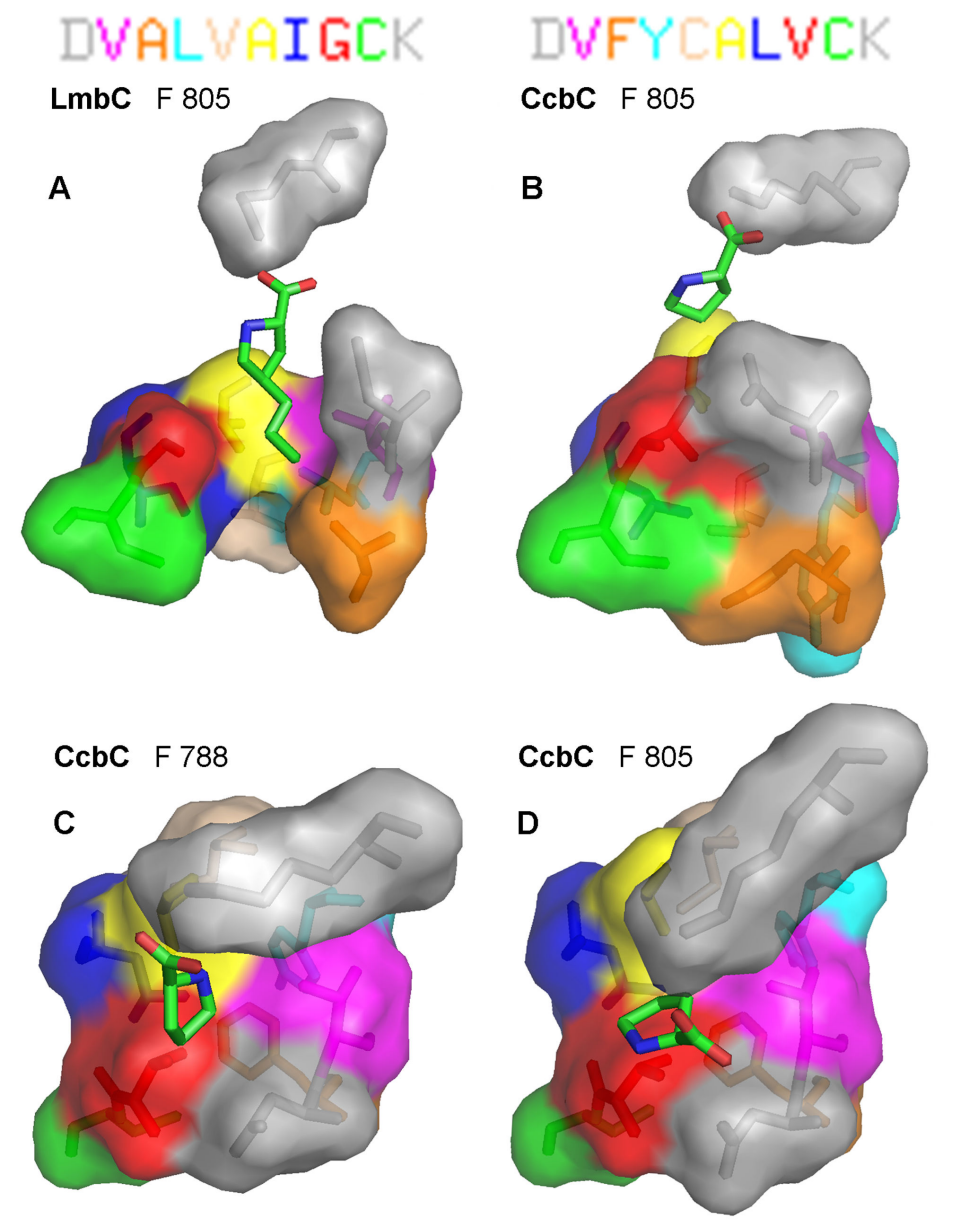
Homology models of the CcbC and LmbC binding pocket with the substrate. The models of LmbC (A) and CcbC (B) at frame 805 (time 8.05 ns) of a 20-ns-long, non-restrained MD simulation. Pictures C and D at frame 788 (7.88 ns) and frame 805 (time 8.05 ns) represent another perspective of the CcbC homology model. The letters of the nonribosomal code at upper edge are colored to correspond to the individual amino acid residues of the structures.

LmbC and CcbC homology models were constructed based on the structure of the phenylalanine specific A-domain of GrsA (called PheA, PDB ID 1AMU) which has bound AMP and Phe. A model of the LmbC G308V point mutant was generated by *in silico* mutation of G308 in the LmbC model. The L-proline (in CcbC) and PPL (in LmbC and LmbC G308V) substrates were positioned by superimposing the models on the PheA structure and refined according to the location of the amino and carboxylate groups of the bound L-phenylalanine in the PheA structure. CcbC and LmbC have quite low homology to PheA (26.4% and 24.9%, respectively). In these situations, there is always a danger that the resulting homology model may violate the ordinary parameters of real proteins. In order to produce a reliable model, 20-ns-long MD simulations were employed to relax any and all possible strains that may have arisen from model building. The relaxed models of LmbC and CcbC from frame 805 corresponding to time 8.05 ns are presented in Figure 4A and B. Time-based and residue-based root-mean-square deviation (RMSD) analyses confirmed the general overall stability of the LmbC and LmbC G308V models during the whole simulation period (for details see Analysis S1). However, during the second half of the production phase, the RMSD values of the CcbC model increased and fluctuated, indicating that this model is of only limited validity. A residue-based RMSD analysis of this model at the beginning and end of the MD simulation confirmed several flexible regions. Fortunately, none of the ten amino acid residues forming the CcbC substrate binding pocket belong to any of these regions, indicating that the CcbC substrate binding pocket remained a compact and stable structure during the whole simulation period. Additionally a longer 100-ns-long MD simulation was performed to confirm the stability of the LmbC and CcbC models. Time-based RMSD analysis of both models showed the convergence and thus the stability of the system (not shown).

### Evolution of the CcbC and LmbC substrate binding pocket architecture and function

Time-based RMSD analyses of all substrate C atoms during the 20-ns-long MD simulations were performed to evaluate the substrate’s conformation and its interactions with the CcbC and LmbC binding pockets (Figure S4G). For LmbC (Figure S4D–F and blue line in S4G), the PPL substrate remained in a stable position and in the correct orientation in the substrate binding pocket during the simulation period. Its RMSD fluctuated only slightly around a mean value of ~1.5 Å (±0.5 Å). Although L-proline also remained in contact with the substrate binding pocket in the CcbC model (Figure S4A–C and green line in S4G), its mean RMSD reached ~5 Å during the second part of the production phase and exhibited substantial fluctuations (±1 Å). Thus, instead of being strongly fixed, L-proline shifts and rotates in the CcbC binding pocket. This is shown more clearly in Figure 4C-D, where the L-proline rotates in the substrate binding pocket during a 0.17 ns period in the middle of the production phase (frames 788 and 805, between 7.88 and 8.05 ns). This is likely a further indication of the limited validity of the CcbC model. Moreover during a longer 100-ns-long MD simulation the L-proline substrate was released from the CcbC substrate binding pocket after ~40 ns, whereas PPL remains in substrate binding pocket of LmbC for the whole production phase (not shown).

**Figure 5:**
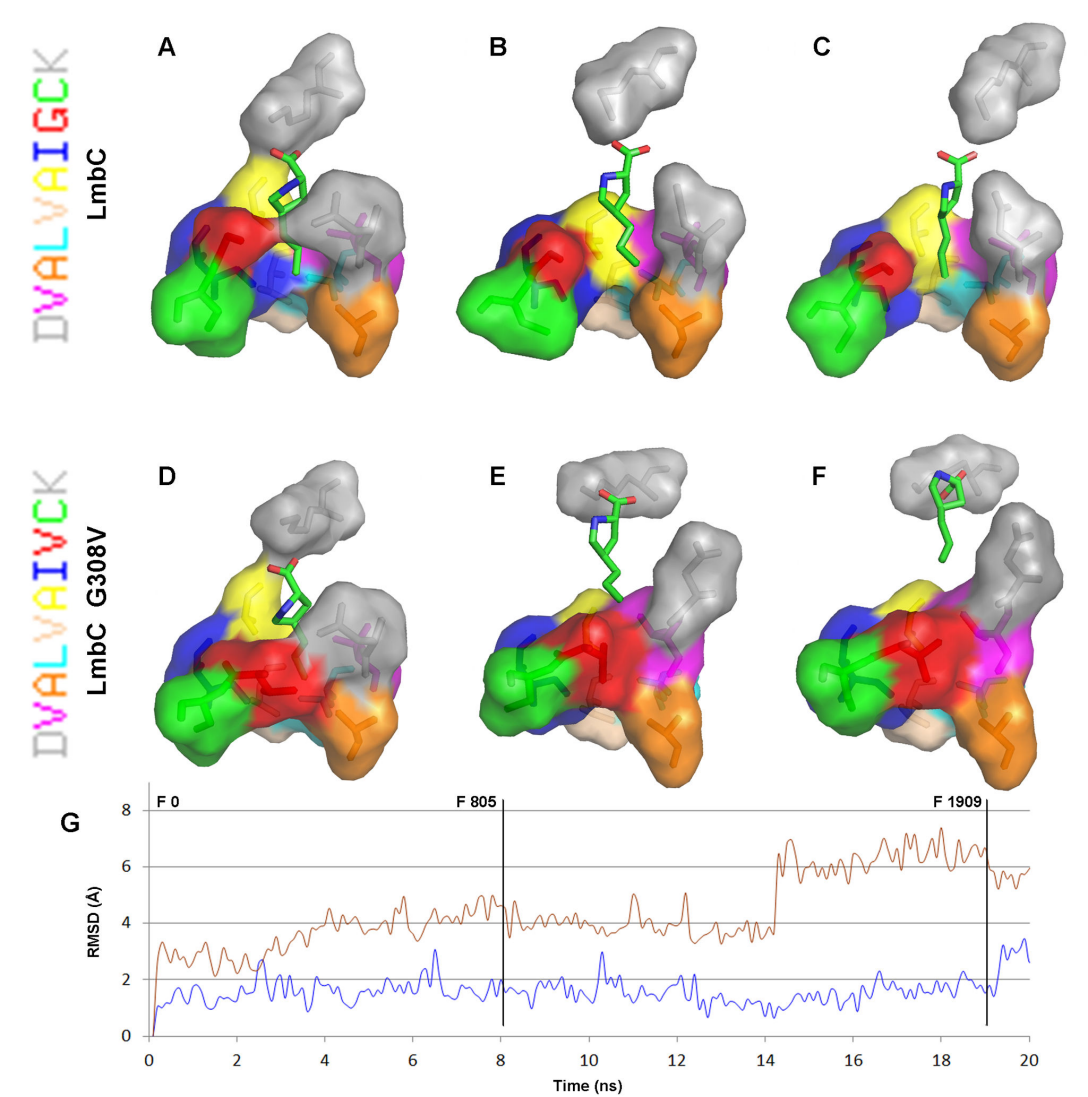
Homology models of the LmbC and LmbC G308V amino acid binding pocket and an RMSD analysis of these models during MD simulations. Structures of the substrate binding pockets from LmbC (A–C) and LmbC G308V (D–F) homology models with bound PPL during the course of a 20-ns-long, non-restrained MD simulation are shown at 0 ns (left column), 8.05 ns (middle column), and 19.09 ns (right column). The nonribosomal code of each model is displayed at left. The individual letters of the code are colored to correspond to those of the individual amino acids in the structures. A time-based RMSD analysis of the substrate during a 20-ns-long, non-restrained MD simulation of LmbC (blue line) and LmbC G308V (red line). The RMSD was calculated over all substrate C atoms. The positions of the frames 0, 805 and 1909 (corresponding to the time 0 ns, 8.05 ns, and 19.09 ns) are marked with vertical lines.

Two amino acid residues of all A-domain substrate binding pockets are highly conserved, a L-lysine which interacts with the carboxylate group, and an L-aspartate which interacts with the α-amino group of the substrate amino acid (colored gray in Figures 4, 5 and S4) [45,46]. All these weak interactions were proved during MD simulations of LmbC and CcbC models with the only exception of α-amino group of L-proline and D201 of CcbC reflecting the above mentioned rotation of the substrate.

The remaining eight amino acids determine the substrate specificity of the A-domain (colored variously in Figures 4, 5 and S4). As Figure 4 shows, the substrate binding pocket of CcbC clearly has a smaller, tighter cavity. In addition to the invariant residues D201 and K490, the L-proline substrate is in direct contact only with three amino acids: V202, A274 and V306. The remaining five residues of the nonribosomal code are sterically screened by these three (and likely serve to maintain the overall shape of the binding pocket). Similarly formed binding pockets, in which only a few residues of the nonribosomal code directly participate in substrate binding, have previously been described for other A-domains [53-55]. The CcbC homology model clearly accounts for the inability of the protein to accept and activate L-proline derivatives with any side chain in position 4 (Figure 4B–D): The binding site is both too small and the wrong shape to accommodate the side chain.

The nonribosomal code of the LmbC substrate binding pocket differs from that of CcbC in five of the eight residues determining the substrate specificity. These differences result together in a formation of the channel accommodating the alkyl side chain of PPL. Interestingly, only one of the three residues which directly contact the substrate in the CcbC binding pocket is altered in LmbC: V306 in CcbC has become G308 in LmbC (red in Figures 4B–D). The change from L-valine to glycine seems to be critical; the larger L-valine should block the accessibility of the channel for the PPL alkyl side chain. The model shows that the other four differences also contribute to the formation of a channel of a proper size, shape and hydrophobicity (Figure 4A). A207 in LmbC in contrast to F205 in CcbC makes the channel more spacious (orange residue in Figure 4A), whereas more hydrophobic L246 and V274 (LmbC) in contrast to Y244 and C272 (CcbC) correspond better to the accommodation of the hydrophobic alkyl side chain of PPL. The MD simulation indicates that the PPL substrate is considerably better anchored in the LmbC binding pocket than L-proline in the CcbC pocket (Figure S4G, blue vs. green line), most likely due to an increased number of contacts between the substrate and the enzyme. This feature of the model agrees with the observed kinetic parameters of CcbC and LmbC. The substrate affinity, measured by *K*_m_, increases with the length of the substrate’s alkyl side chain: The *K*_m_ for L-proline bound to CcbC is 0.5 ± 0.03 mM, 0.29 ± 0.03 mM for PPL bound to LmbC, 0.118 ± 0.008 mM for BuPL bound to LmbC, and 0.0596 ± 0.003 mM for PePL bound to LmbC.

### *In silico* and biochemical analysis of mutant LmbC G308V

Homology models of LmbC/CcbC indicated the importance of the residues G308/V306. Two homology models of mutant LmbC G308V and CcbC V306G were constructed. From the CcbC V306G model it appears that the glycine residue itself in this position does not ensure formation of a channel of the appropriate size and shape to accommodate the alkyl side chain of PPL. It corresponds with the fact that the nonribosomal codes of all known A-domains recognizing the 2C and 3C branched L-proline derivatives differ from the L-proline consensus in 3-6 amino acid residues. On the other hand the LmbC G308V model suggests that L-valine in this position can efficiently block the channel. The glycine residue at the tested position thus seems to be necessary, but not sufficient to harness branched L-proline derivatives. In order to test the outputs of the homology models, i.e. the existence of the channel accommodating the alkyl side chain of PPL in LmbC, the above mentioned LmbC G308V mutant was tested both *in silico* and biochemically.

The time-based RMSD analysis of all PPL C atoms during a 20-ns-long MD simulation was carried out to evaluate the substrate interactions with the substrate binding pocket of LmbC G308V. The divergent character of the time-based RMSD plot of PPL bound to LmbC G308V (Figure 5D–F and red line in 5G) does not reflect a poor homology model used here, but rather indicates a real incompatibility of the enzyme-substrate pair. The RMSD increases in three steps during the MD simulation: A mean value of ~3 Å for 0–4 ns, ~4 Å for 4–14 ns and ~6 Å for 14–20 ns. The movement of the substrate out of the binding pocket is clearly seen in Figure 5D–F. At the beginning of the simulation, the PPL substrate was buried inside the substrate binding pocket, as in the wild type LmbC. After the substrate moved out of the binding pocket a conformational change occurred, which made the pocket inaccessible, similar to the situation in CcbC. As a result, the channel for the PPL alkyl side chain disappeared. This simulation suggested that much worse kinetic parameters can be expected for the reactions of LmbC G308V with substrates containing an alkyl side-chain compared to the wild-type LmbC.

To test these predictions experimentally, the LmbC G308V mutant form was constructed by site-directed mutagenesis, and overproduced, purified and assayed under the same conditions as LmbC. The kinetic parameters for the reaction of this mutant protein with PPL, EPL and L-proline are shown in Figure 3G–I and summarized in Table 1. The LmbC G308V mutant exhibited a ~20× higher *K*_m_ and a ~100× lower *k*_cat_, resulting in a ~2x10^3^-fold lower catalytic efficiency for PPL compared to the wild-type LmbC. Similarly, the kinetic parameters for EPL also worsened dramatically. On the other hand, the *k*_cat_/*K*_m_ ratio for L-proline is almost unaffected by this mutation. Although the affinity (*K*_m_) to the L-proline substrate may be slightly better in the mutant form (260 ± 20 vs. 470 ± 60 mM), the catalytic efficiency declined reciprocally (0.019 vs. 00.043 mM^-1^min^–1^). It should be noted that the measured parameters probably reflect a combination of two independent factors. Namely, the above mentioned selective response and, to a minor extent, a nonselective worsening of the overall catalytic efficiency of the mutant protein. This is a common consequence of artificial changens of natural proteins. In summary, this single G308V point mutation abolishes LmbC’s natural preference for PPL, making thus PPL not much better than L-proline as the substrate. The results of these biochemical assays fully confirmed those predicted by the simulation.

Among the modular A-domains activating branched L-proline derivatives, only HrmP(3)_A_ from hormaomycin biosynthesis seems to have followed a similar mechanism. Compared to the consensus pattern of the L-proline specific modular A-domains, the code of HrmP(3)_A_ has three substitutions, all of them for smaller residues rather than larger ones. Also, similar to LmbC, HrmP(3)_A_ has a glycine in the position corresponding to the residue 308 instead of a consensual L-valine. Presumably, this substitution plays the same role as in LmbC, namely to facilitate the access of a substrate alkenyl side chain inside the binding pocket. Analogously to the evolution of LmbC, also the other two substitutions in the HrmP(3)_A_ substrate binding pocket, V to A and I to S, could co-operate in the formation of a channel accommodating the alkenyl side-chain of hormaomycin precursor. Recently [56], HrmP(3)_A_ was biochemically proved to adenylate its putative natural substrate (2*S*,4*R*)-4-(*Z*)-propenyl-L-proline. Kinetic parameters have not been estimated but the protein highly preferred the branched derivative over L-proline and other tested amino acids.

## Conclusion

### General aspects of the evolution of PPL biosynthesis and incorporation

The term “specialized metabolism” is currently often used instead of “secondary metabolism” [57,58] in order to emphasize the essence: more active derivatives can arise from unique, i.e. specialized, biosynthetic pathways and, even better, from their combinations. The biosynthesis of complex natural compounds is encoded by biosynthetic gene clusters which contain subclusters, groups of genes coding for individual specialized building units of the final product. The HGT and a fusion of subclusters to produce new or more complex gene clusters is generally known as a common mechanism in the evolution of biosynthesis of new secondary metabolites [59]. The most puzzling seems to be the evolution of genes coding for the enzymes linking the structural blocks together. Such condensing enzymes are necessary for the functioning of a new fusion cluster, but were not required for the ancestral gene clusters. Thus, their evolutionary origin is unclear [59,60]. Clearly, the genesis and evolution of the condensation enzymes, particularly their substrate specificity adaptation to newly emerging intermediates, seem to be a key element for understanding how new biosynthetic clusters for secondary metabolites arise.

The 2C and 3C branched L-proline derivatives are highly specialized building blocks integrated as precursors exclusively in several PBDs, lincomycin and hormaomycin. Logically, a coupled HGT of genes coding for both the biosynthetic and integration steps appears to be the simplest evolutionary mechanism, probably involved in the evolution of PBD compounds exhibiting high structural variability of incorporated building blocks but sharing an identical overall core structure [61]. A HGT of a whole biosynthetic cluster including genes coding for the integrating NRPSs followed by point mutations of modular A-domains was the most probable mechanism of structural diversification of PBD compounds.

The NDLS, catalyzing a condensation of building units in the biosynthesis of lincosamides, functionally differs from typical modular NRPSs operating in the biosynthesis of PBDs and hormaomycin: NDLS attaches the activated amino acid to the amino sugar, but not to another amino acid, unlike the “authentic” NRPS. This is probably the most interesting aspect of evolution of lincomycin biosynthesis. The ancestral NDLS represents a typical example of a specialized condensing enzyme realizing a connection of two types of building units: one specialized metabolite, an amino sugar, and one primary metabolite, proteinogenic L-proline. In the lincomycin biosynthesis, moreover, the NDLS A-domain LmbC was adapted from using L-proline to a new unusual specialized metabolite, PPL, giving rise to unique connecting functionality. This new condensing activity is distinct from those found in both ancestral clusters. From the point of view of the PPL donor biosynthetic cluster, the NDLS attaches the precursor to a novel type of building unit (amino sugar instead of an original amino acid). From the point of view of the acceptor biosynthetic cluster, the adaptation led to the biosynthesis of a more complex compound combining two specialized building units.

In the final step of lincomycin biosynthesis the amino acid moiety of the NDL is N-methylated by LmbJ. A wide variety of modifications was described in PBDs, however the N-methylation of L-proline derived building unit is lincosamide specific and arose from the ancestral biosynthetic cluster. The N-methylation step was preserved also in the newly evolved lincomycin biosynthetic cluster due to the relaxed substrate specificity of the *N*-methyltransferase enzyme [49].

## Experimental Section

### Construction of LmbC and CcbC expression vectors

The *lmbC* gene was PCR amplified from the chromosomal DNA of the lincomycin producing type strain *Streptomyces lincolnensis* ATCC 25466. The *ccbC* gene was PCR amplified from a SuperCos cosmid vector I (Stratagene) carrying a fragment of the celesticetin gene cluster from the celesticetin producing type strain *Streptomyces caelestis* ATCC 15084; GenBank GQ844764.1. The following primers were used for *lmbC*: lmbCf 5’-CGAATTCCATATGTCGTCCTCCGTTCGA-3’ and lmbCr 5’-CCGCTCGAGCTCCCCGCGTGTGACGA-3’ (the *Nde*I and *Xho*I restriction sites are underlined). For *ccbC*, the following primers were used: CCF 5’-CCGGAATTCCATATGAATACCTCCACTGTCCG-3’ and CCRN 5’-AACCCAAGCTTACAGCGTGACGTACCG-3’ (*Nde*I and *Hind*III restriction sites are underlined). The *lmbC* gene was inserted into pET42b (Novagen) via the *Nde*I and *Xho*I restriction sites. The resulting plasmid plmbC1 was used to produce a C-terminally His_8_-tagged LmbC. The *ccbC* gene was inserted into pET28b (Novagen) via the *Nde*I and *Hind*III restriction sites. The resulting plasmid pccbC was used for the production of an N-terminally His_6_-tagged CcbC. The open reading frames of both genes were confirmed by sequencing.

Recombinant LmbC with an N-terminal His-tag was also produced, but co-expression with GroES and GroEL chaperonins was required to produce soluble protein. During the isolation step, it was not possible to separate LmbC from GroEL. Both the N- and C-terminally His-tagged proteins exhibited the same activities when assayed; consequently, C-terminally His-tagged LmbC was used in the present study.

### Site-directed mutagenesis of *lmbC* in plmbC1

The *lmbC* gene was excised via the *Nde*I and *Xho*I restriction sites from plmbC1 and inserted into a pJAKO [62] cloning vector, derived from pBluescript II KS+ (Stratagene) using the same restriction sites. The resulting plmbC2 plasmid was used for *in vitro* site-directed mutagenesis using the QuickChange Site-Directed Mutagenesis Kit (Stratagene) and mutagenic primers G308Vf 5’-CAACATCTAC GGTCCGACCGAGACCAACG**T**CTGTACGTACG-3’ and G308Vr 5’-CGTACGTACAGACGTTGGTCTCGGTCGGACCGTAGATGTTG-3’. The point mutation G923T, which codes for an L-valine residue rather than an glycine at position 308 in LmbC, is in bold in the forward primer. A silent C906T mutation, introducing a TCCGAC *Mme*I restriction site to verify the mutation, is italicized and underlined. The sequence of the reverse primer was the reverse complement of the forward primer. The resulting plmbC3 plasmid was digested with *Nde*I and *BsiW*I and the mutated segment of the *lmbC* gene (934 bp) was inserted into the vector plmbC1 instead of the non-mutated segment using the same restriction sites to produce plmbC4. The sequence of this plasmid was confirmed by sequencing and used in the production of a C-terminally His-tagged LmbC G308V mutant.

### Expression and purification of LmbC, LmbC G308V and CcbC

The His-tagged LmbC, LmbC G308V and CcbC were produced in *E. coli* BL21(DE3) (Novagen), transformed by plmbC1, plmbC4 or pccbC, as appropriate. LB medium (0.1 L) with kanamycin (30 mgL^–1^) was inoculated and grown at 37°C. At OD_600_ = 0.7, the culture was cooled down to 17°C and the overexpression was induced by 0.4 mM isopropyl-β-D-thiogalactopyranoside. Cells were grown for an additional 20 hours at 17°C, harvested by centrifugation and stored frozen at –20°C.

Proteins were purified from crude cell extracts, which were prepared by ultrasonic homogenization in TS-8 buffer (20 mM Tris-HCl, 100 mM NaCl, pH 8.0), using HiTrap™ Chelating HP Columns (GE Healthcare). The His-tagged proteins were eluted by TS-8 buffer with 250 mM imidazole. Pooled fractions containing the purified proteins were dialyzed overnight against 50 mM Tris-HCl (pH 8.7) and stored at –20°C. Protein concentration was measured by the Bradford protein assay kit (Bio-Rad) with bovine serum albumin as a standard.

### Enzyme activity assay

Enzyme activity was assayed by amino-acid-dependent exchange of radioactivity from [^32^P]-labeled PPi into ATP. [^32^P]Tetrasodium pyrophosphate was purchased from PerkinElmer. The ATP-[^32^P]PPi reaction mixtures contained 100 mM Tris-HCl (pH 8.7), 2 mM MgCl_2_, 1 mM DTT, 1 mM ATP, 1 mM [^32^P]PPi (400000 CPM) and various concentrations of amino acid substrates; the total volume was 100 μL. Reactions were started by the addition of freshly thawed enzyme in final concentrations of 0.05 μM for LmbC and CcbC or 1 μM for LmbC G308V. After 30 min incubation at 28°C, the reactions were quenched by the addition of 0.5 mL of quenching mixture containing 1.6% (w/v) activated charcoal, 4.5% (w/v) tetrasodium pyrophosphate and 3.5% perchloric acid. The quenched mixture was vortexed and pelleted by centrifugation. The charcoal-containing pellet was washed twice with 0.5 mL of the quenching mixture without charcoal, resuspended in 0.5 mL of water, and submitted for liquid scintillation counting using Beckman LS 6500 liquid scintillation counter. The linearity of the reaction velocity in the 30 minute testing range was confirmed. For each enzyme/substrate combination the reactions mixtures identical to that for the highest used substrate concentration were prepared. The reactions were carried out for 0, 5, 10, 20, 25 and 30 minutes. The resulting plot of product formation vs. time showed straight line. The kinetic parameters were determined by non-linear least squares fitting.

### Preparation of (2*S*,4*R*)-4-alkyl-L-prolines

Solvents and reagents were purchased from Sigma-Aldrich. NMR spectra were recorded on a Bruker AVANCE III 400 MHz NMR (400.13 MHz for ^1^H and 100.62 MHz for ^13^C, Bruker BioSpin GmbH, Rheinstetten, Germany) in CDCl_3_ or DMSO-*d*_*6*_ at 30°C. (2*S*,4*R*)-4-Butyl-L-proline and (2*S*,4*R*)-4-pentyl-L-proline were prepared according to a previously described procedure [51]. (2*S*,4*R*)-4-Propyl-L-proline was prepared by alkylation of protected L-pyroglutamic acidusing allyl bromide, followed by a two-step reduction of the resulting amide to yield protected 4-allyl-L-proline. Protected 4-allyl-L-proline was then hydrogenated using a standard H_2_-Pd/C procedure, affording, after subsequent deprotection steps, the final product (2*S*,4*R*)-4-propyl-L-proline. The preparation of (2*S*,4*R*)-4-ethyl-L-proline was based on the controlled condensation of a protected derivative of L-pyroglutamic acid with acetaldehyde. The resulting aldol was dehydrated using MsCl/Et_3_N, affording 4-ethylidene-pyroglutamate. After hydrogenation (Pd/C), this gave *cis*-3,5-disubstituted 2-pyrrolidone. Inversion of configuration at pyroglutamate C-4 led to trans-3,5-disubstituted 2-pyrrolidone, which, after subsequent reduction of amide to amine and deprotection, led to the final product (2*S*,4*R*)-4-ethyl-L-proline. For details of the synthetic procedures and analysis of the products, see Supporting information.

### Phylogenetic analysis

The closest homologs of LmbC and CcbC were found using a blastp search at the NCBI web site (http://blast.ncbi.nlm.nih.gov/Blast.cgi). The amino acid sequences of the A-domains were retrieved from GenBank. Sequence identities of these proteins to LmbC and CcbC were calculated in Geneious 5.5.6 [63] based on pairwise alignments generated using MAFFT software version 7.037b at the CBRC web site (http://mafft.cbrc.jp/alignment/server) [64]. For these alignments, the full length sequences of the stand-alone A-domains (exception: the last 64 amino acids of RphM were removed) and those of just the relevant A-domains extracted from the sequences of modular NRPSs to match the length of LmbC and CcbC were used.

To construct the phylogenetic trees, a multiple sequence alignment was generated using MAFFT and a neighbor-joining and maximum likelihood phylogenetic trees were constructed using MEGA5 version 5.2 [65]. The sequence of acetyl-CoA synthetase was used as the outgroup; bootstrapping was performed with 100 replications.

### Homology model construction

The structure of the L-phenylalanine specific A-domain of GrsA (also called PheA, PDB ID 1AMU) was used as a template for the construction of both LmbC and CcbC homology models. To build the models, the sequences of LmbC and CcbC were aligned to 1AMU using the ClustalX version 2.0.10 [66]. Model structures were produced using the SWISS-MODEL server (http://swissmodel.expasy.org) in alignment mode [67]. The modeling software did not incorporate the two C-terminal residues of both proteins into the final models. Model of LmbC G308V was generated by *in silico* mutation in model of LmbC. The positions of AMP, Mg^2+^, and the amino acid substrates in these models were determined by superimposing the models on the PheA template in PyMOL [68] and adjusting the positions of PPL and L-proline based on the positions of the α-amino and carboxylate groups of the L-phenylalanine bound to PheA.

### Molecular dynamics simulations

All molecular dynamics (MD) simulations were carried out using the AMBER suite [69] with the parm99SB force field [70]. The following simulation protocol was used: First, the protonation states of all L-histidine residues were set to create an optimal H-bond network. Next, all remaining hydrogen atoms were added using the Leap program from the AMBER package. Then the structures were charge-neutralized by adding an appropriate number of Na^+^ ions. To prevent rotation of the entire molecule, the center of mass and the orientation of the protein were fixed. All systems were inserted into a rectangular water box filled by TIP3P water molecules; the layer of the water molecules was 9 Å thick. Each system was then minimized in the following way: The protein was frozen while the solvent molecules and counter ions were allowed to move during a 1000-step minimization process, followed by a 10-ps-long MD run under NpT conditions (i.e. p = 1 atm, T = 298.15 K). The side chains were then relaxed by several sequential minimizations with decreasing force constants applied to the backbone atoms. After relaxation, the system was heated to 50 K for 20 ps and then up to 298.15 K for 90 ps. The particle-mesh Ewald method for treating electrostatic interactions was used. For the production phase, all simulations were run under periodic boundary conditions in the NpT ensemble at 298.15 K and at a constant pressure of 1 atm using a 2-fs time integration step. The SHAKE algorithm with a tolerance of 10^–5^ Å was applied to fix all bonds containing hydrogen atoms. A 9.0 Å cutoff was used to treat non-bonding interactions. Coordinates were stored every 10 ps (i.e. 100 frames correspond to 1 ns of simulation). The total duration of each production phase, along with the total numbers residues, atoms, counter ions and water molecules in each of the systems studied, are shown in Table 2.

**Table 2:** Production phases parameters used in MD simulations.

**Protein**	**No. of residues**	**Ligand**	**Na^+^**	**No. of H_2_O molecules**	**No. of atoms**
LmbC	507	PPL	6	18240	62238
LmbC G308V	507	PPL	6	17156	58995
CcbC	503	L-proline	14	18746	63739

Total duration of all MD simulations was 20 ns; 1 molecule of AMP and 1 Mg^2+^ ion were present in all models.

### RMSD analysis of MD simulations

Time-based and residue-based RMSDs were used to monitor trajectory stability and conformational changes. For time-based analysis, RMSDs between the starting and current structures were calculated in 0.1 ns intervals during the whole production phase of the MD simulation. The RMSDs of the protein models were calculated using only the backbone Cα atoms while substrate RMSDs (for PPL and L-proline) were calculated using all substrate C atoms. For residue-based analysis, RMSDs were calculated for every residue between the starting and final structures at the end of the MD simulation. The MDTRA software package was used for all RMSD calculations [71]. All protein structure figures were generated by PyMOL [68].

## Supporting Information

Analysis S1 **Verification of homology model structure by MD simulation.** (DOCX)

Figure S1 **Phylogenetic trees of A-domains specific for L-proline or its derivatives.** Rooted, neighbor-joining (A) and maximum likelihood (B) phylogenetic trees were constructed based on the full length amino acid sequences of stand-alone A-domains and the excised sequences of modular A-domains. Bootstrap values (100 replicates) above 50 % are indicated at the nodes. The names of A-domains are identical to those in Figure 2. The horizontal bar indicates the number of amino acid substitutions per site.
(TIF)

Figure S2 **Comparison of the nonribosomal codes of A-domains activating L-proline and L-proline derivatives.** The highly conserved D and K residues at the boundaries of nonribosomal codes are omitted. The same set of A-domains is shown as in Figure 2. Amino acids are numbered at the top according to the A-domain of GrsA (PheA) (first row), CcbC (second row), and LmbC (third row). Substrates are abbreviated as in Figure 2. Residues of stand-alone A-domains in accordance with consensus of L-proline specific stand-alone A-domains are in blue. Residues of modular A-domains in accordance with consensus of L-proline specific modular A-domains are in red. *Number in parentheses behind the name of respective NRPS denotes the number of the module in NRPS protein chain, if relevant; letter in parentheses denotes the source organism.
(TIF)

Figure S3 **SDS PAGE analysis of purified CcbC, LmbC and LmbC G308V proteins.** **12% (w/v) gel**. Arrow shows the position of purified proteins. Lanes: S - PageRuler^TM^ prestained protein ladder; 1 - His-tagged CcbC; 2 - His-tagged LmbC; 3 - His-tagged LmbC G308V.
(TIF)

Figure S4 **Homology models of the CcbC and LmbC amino acid binding pocket and an RMSD analysis of these models during MD simulations.** Structures of the substrate binding pockets from CcbC (A–C) and LmbC (D–F) homology models with bound substrates during the course of a 20-ns-long, non-restrained MD simulation are shown at 0 ns (left column), 8.05 ns (middle column), and 19.09 ns (right column). The nonribosomal code of each model is displayed at left. The individual letters of the code are colored to correspond to those of the individual amino acids in the structures. L-proline substrate was used in the CcbC structures while PPL was used for the LmbC models. (G) A time-based RMSD analysis of the substrates during a 20-ns-long, non-restrained MD simulation of CcbC with L-proline (green line) and LmbC with PPL (blue line). The RMSD was calculated over all substrate C atoms. The positions of the frames 0, 788, 805 and 1909 (corresponding to the time 0 ns, 7.88 ns, 8.05 ns, and 19.09 ns) are marked with vertical lines.
(TIF)

Table S1 **Overview of all tested combinations (A-domain vs. substrate) by biochemical assay.** (PDF)

Protocol S1 **Preparation of (2*S*,4*R*)-4-alkyl-L-prolines.** (PDF)
