# Supplementary material for: Adaptation of an L-Proline Adenylation Domain to Use 4-Propyl-L-Proline in the Evolution of Lincosamide Biosynthesis
Source: PLoS One. 2013 Dec 27;8(12):e84902. doi: 10.1371/journal.pone.0084902 (PMC3874040; doi:10.1371/journal.pone.0084902)
Supplement: Analysis S1 — Verification of homology model structure by MD simulation. (DOCX) [file pone.0084902.s001.docx]

**Verification of homology model structure by MD simulation**

The stability of the structures of the homology models of CcbC, LmbC and LmbC G308V were verified by a 20-ns-long, non-restrained MD simulation. Time-based and residue-based RMSDs were used to monitor the overall stability and conformational changes of these proteins. The time-based RMSDs (Figure S5A) calculated over all protein backbone C*α* atoms show a slight conformational relaxation in all three models. The beginning of the production phase is characterized by an increase of RMSD up to ~2 Å. From 3–10 ns, the RMSDs fluctuated slightly between 2.4–3.2 Å. During the second half of the production phase, the conformations of the LmbC and LmbC G308V structures remained stable with RMSDs fluctuating around a mean value of 3.4 Å (±0.4 Å) and 2.8 Å (±0.3 Å), respectively. For the CcbC structure, on the other hand, the mean RMSD value gradually increased from ~2.8 Å in the first half of the production phase to ~4.3 Å at the end of the simulation, accompanied by increase in fluctuation. The simulation, therefore, indicates that the model of CcbC is of only limited validity overall. Accordingly, a residue-based RMSD analysis of this model at the beginning and the end of the production phase (Figure S5B) confirmed several flexible regions. None of ten amino acid residues which form the CcbC substrate binding pocket lies in any of these flexible regions, however, so the binding pocket itself was still stable over the course of the production phase and it was still possible to carry out a meaningful analysis of CcbC using this model. It should also be noted that the LmbC and LmbC G308V substrate binding pockets also remained stable during the production phase.


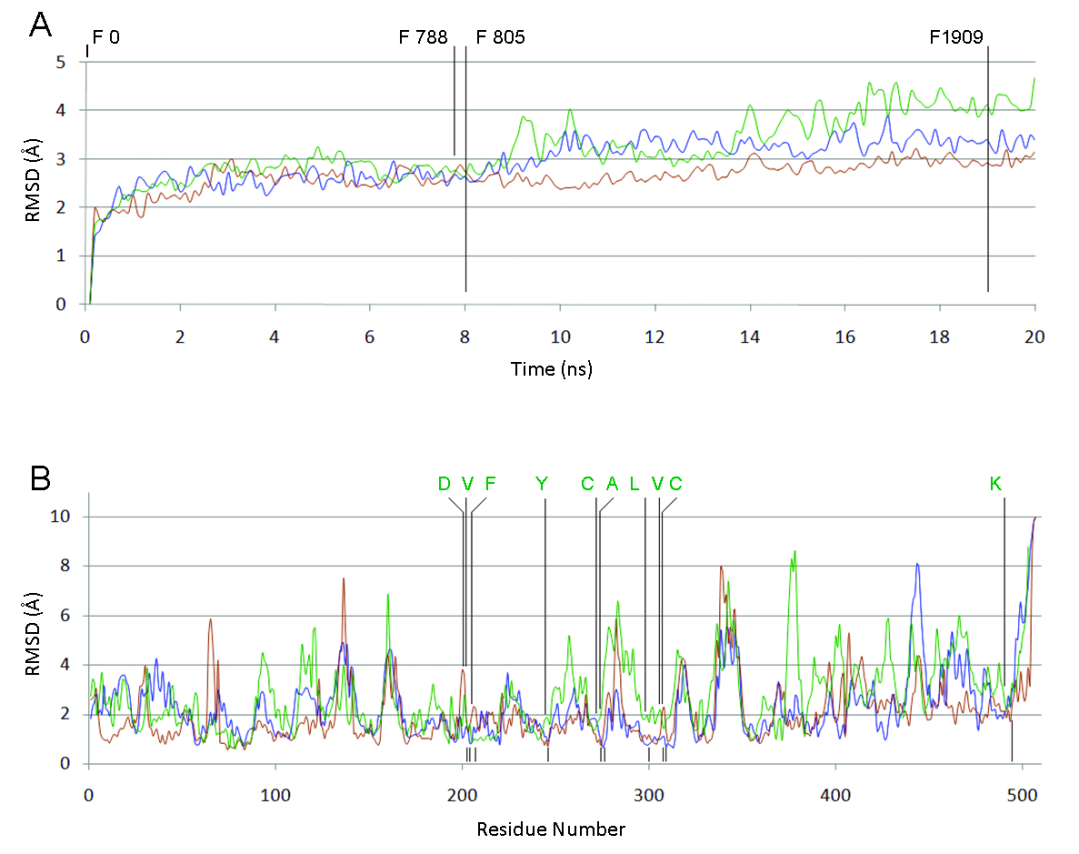


**Figure S5. RMSD analysis of MD simulations.** Time-based (A) and residue-based (B) RMSD analyses of a 20-ns-long, non-restrained MD simulation of CcbC with L-proline (green line), LmbC with PPL (blue line) and LmbC G308V with PPL (red line). The RMSDs were calculated for all backbone C*α* atoms; those of the substrates were not monitored. For time-based analysis (A), the RMSDs were calculated in 0.1 ns intervals during the whole production phase of MD simulation, as a measure of difference between the starting and present structures. For residue-based analysis (B), the RMSDs were calculated for every residue between the starting structure and the structure obtained at the end of the MD simulation. In Panel A, the positions of frames 0, 788, 805 and 1909 (corresponding to time 0, 7.88, 8.05 and 19.09 ns) are marked with vertical lines. The structures of the substrate binding pockets during these frames are shown in Figures 4, 5 and S4. In Panel B, the positions of the amino acid residues which form the nonribosomal code of CcbC are marked by long vertical lines and green letters. The short vertical lines at the bottom of the plot mark the positions of the corresponding residues belonging to the nonribosomal codes of LmbC and LmbC G308V.
