# Supplementary figures and images for: Adaptation of an L-Proline Adenylation Domain to Use 4-Propyl-L-Proline in the Evolution of Lincosamide Biosynthesis
Source: PLoS One. 2013 Dec 27;8(12):e84902. doi: 10.1371/journal.pone.0084902 (PMC3874040; doi:10.1371/journal.pone.0084902)

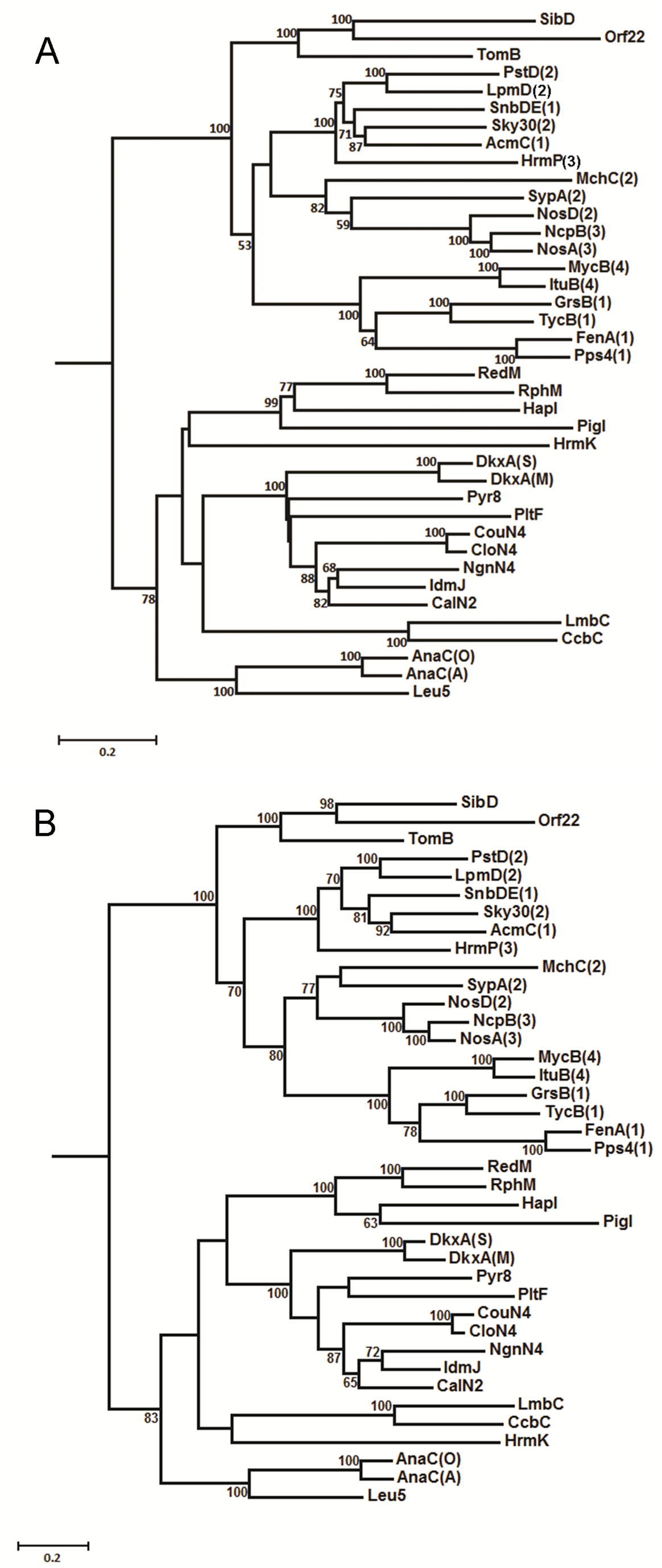

Supplement: Figure S1 — Phylogenetic trees of A-domains specific for L-proline or its derivatives. Rooted, neighbor-joining (A) and maximum likelihood (B) phylogenetic trees were constructed based on the full length amino acid sequences of stand-alone A-domains and the excised sequences of modular A-domains. Bootstrap values (100 replicates) above 50 % are indicated at the nodes. The names of A-domains are identical to those in Figure 2. The horizontal bar indicates the number of amino acid substitutions per site. (TIF) [file pone.0084902.s002.tif]

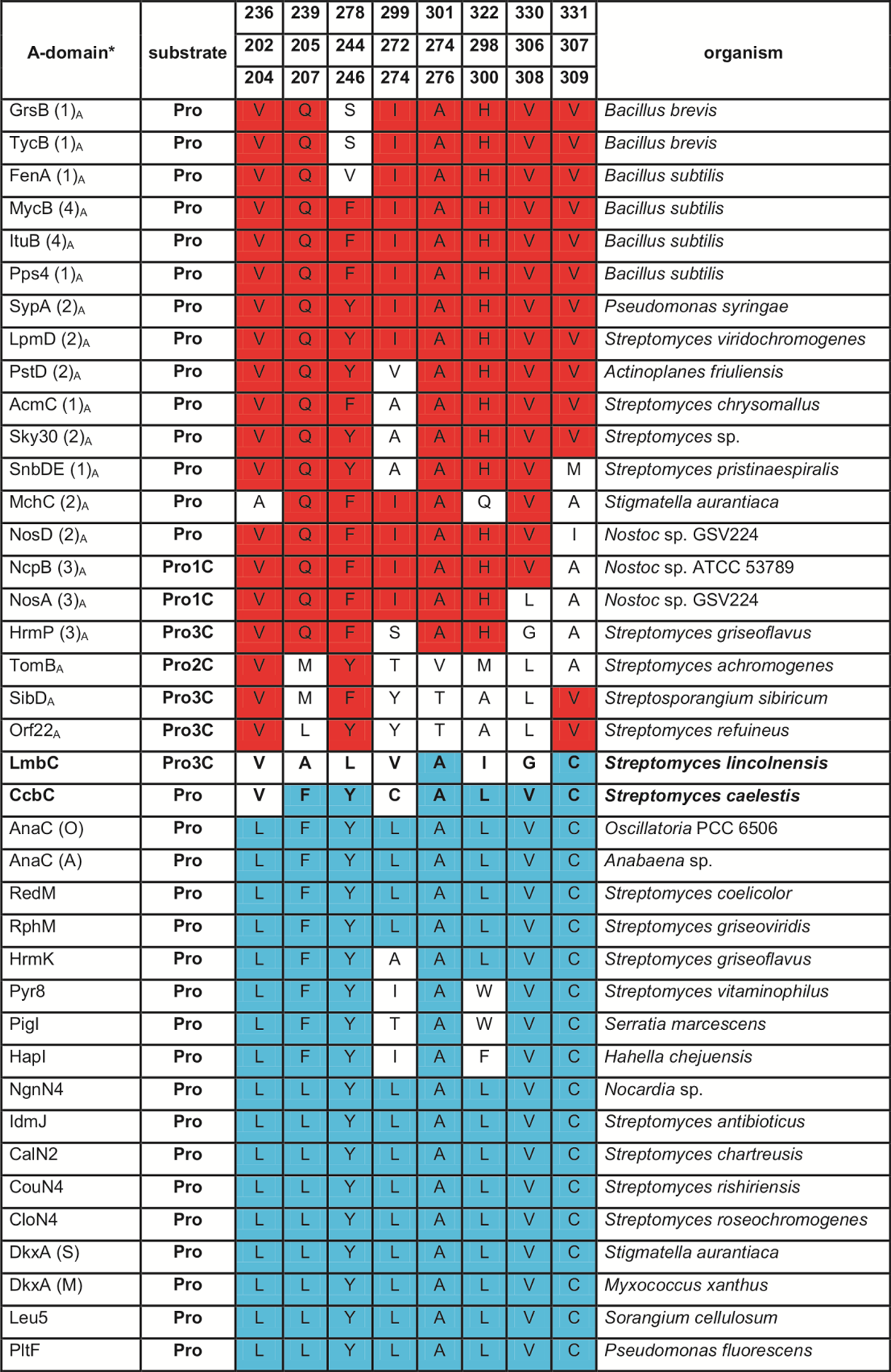

Supplement: Figure S2 — Comparison of the nonribosomal codes of A-domains activating L-proline and L-proline derivatives. The highly conserved D and K residues at the boundaries of nonribosomal codes are omitted. The same set of A-domains is shown as in Figure 2. Amino acids are numbered at the top according to the A-domain of GrsA (PheA) (first row), CcbC (second row), and LmbC (third row). Substrates are abbreviated as in Figure 2. Residues of stand-alone A-domains in accordance with consensus of L-proline specific stand-alone A-domains are in blue. Residues of modular A-domains in accordance with consensus of L-proline specific modular A-domains are in red. *Number in parentheses behind the name of respective NRPS denotes the number of the module in NRPS protein chain, if relevant; letter in parentheses denotes the source organism. (TIF) [file pone.0084902.s003.tif]

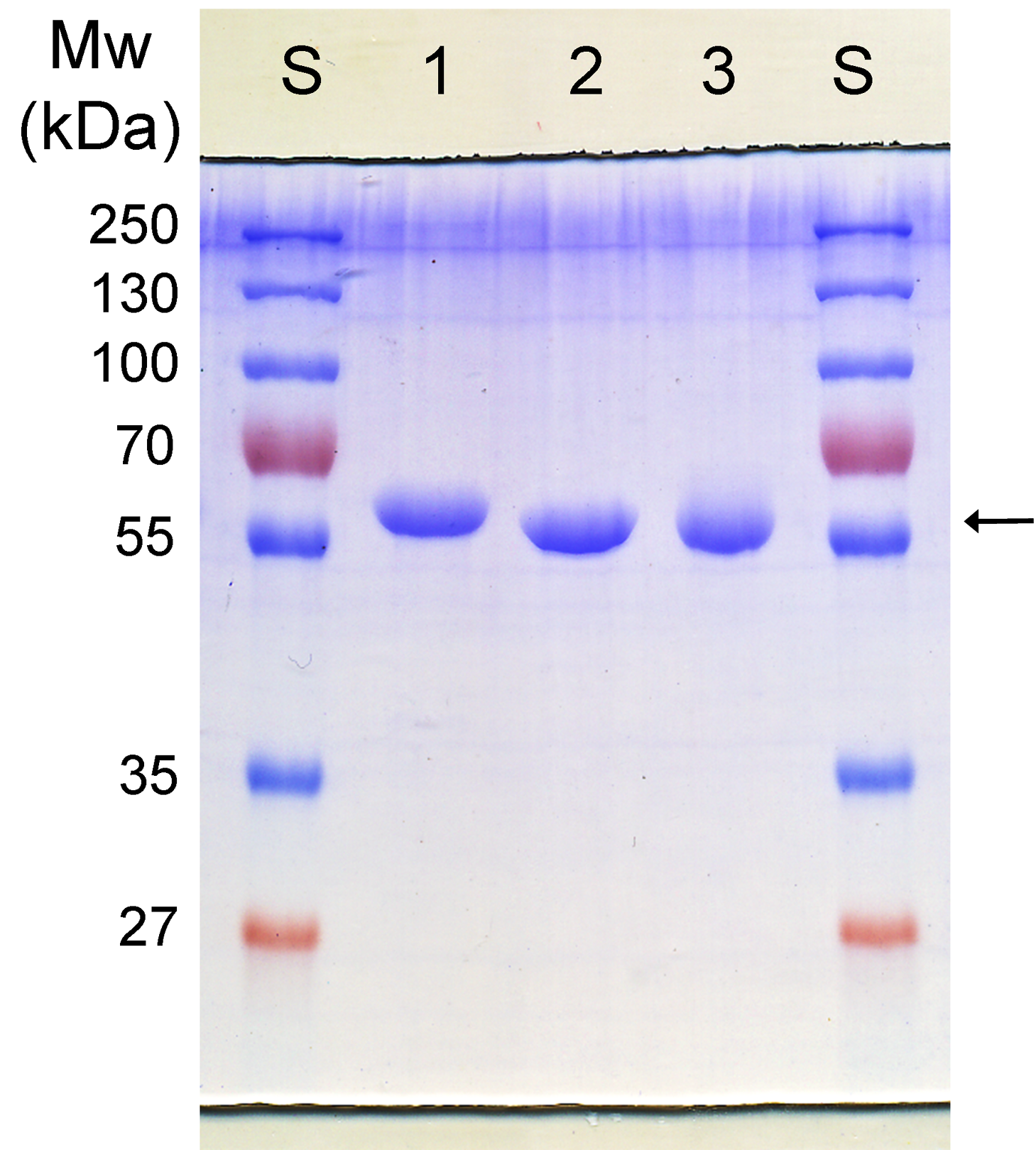

Supplement: Figure S3 — SDS PAGE analysis of purified CcbC, LmbC and LmbC G308V proteins. 12% (w/v) gel. Arrow shows the position of purified proteins. Lanes: S - PageRulerTM prestained protein ladder; 1 - His-tagged CcbC; 2 - His-tagged LmbC; 3 - His-tagged LmbC G308V. (TIF) [file pone.0084902.s004.tif]

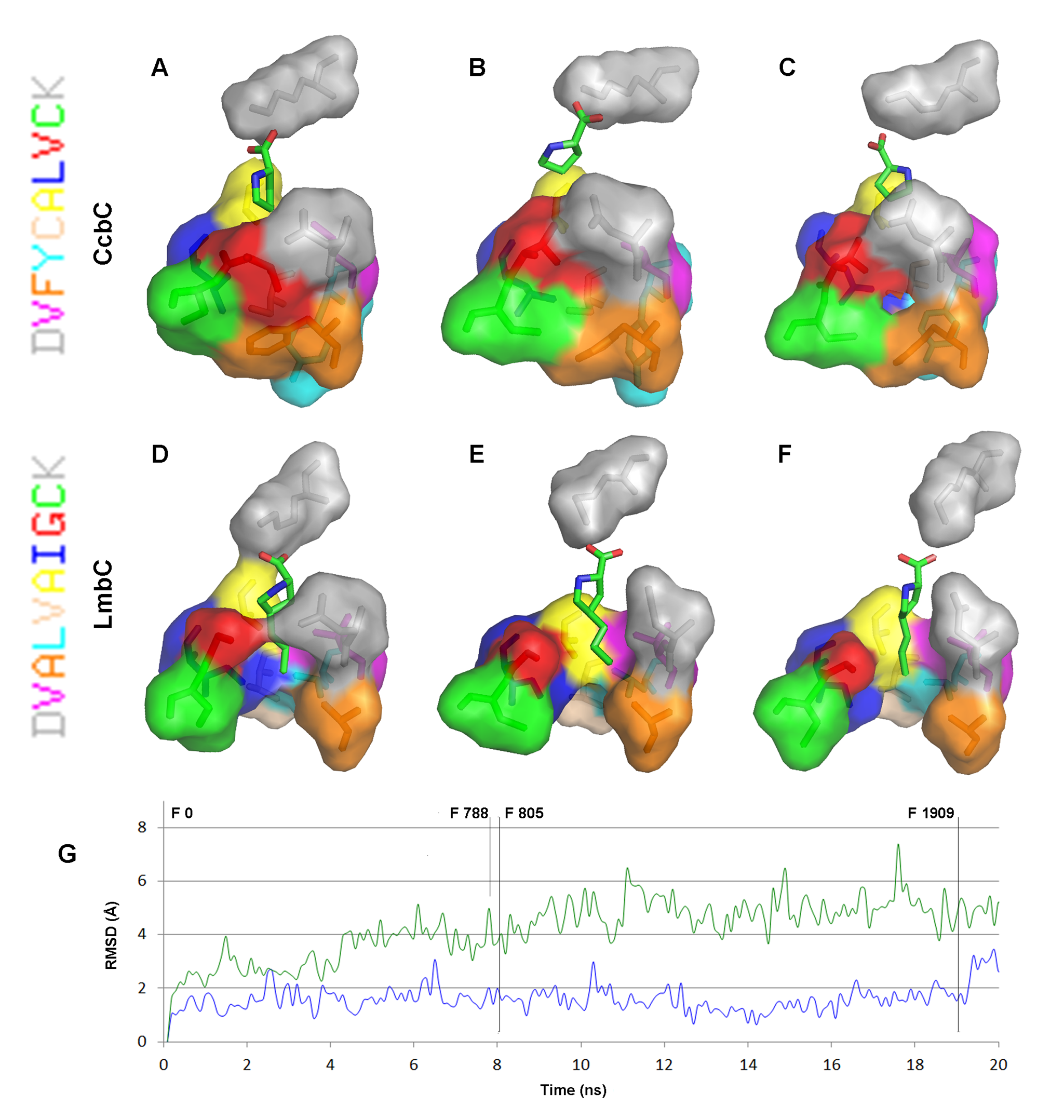

Supplement: Figure S4 — Homology models of the CcbC and LmbC amino acid binding pocket and an RMSD analysis of these models during MD simulations. Structures of the substrate binding pockets from CcbC (A–C) and LmbC (D–F) homology models with bound substrates during the course of a 20-ns-long, non-restrained MD simulation are shown at 0 ns (left column), 8.05 ns (middle column), and 19.09 ns (right column). The nonribosomal code of each model is displayed at left. The individual letters of the code are colored to correspond to those of the individual amino acids in the structures. L-proline substrate was used in the CcbC structures while PPL was used for the LmbC models. (G) A time-based RMSD analysis of the substrates during a 20-ns-long, non-restrained MD simulation of CcbC with L-proline (green line) and LmbC with PPL (blue line). The RMSD was calculated over all substrate C atoms. The positions of the frames 0, 788, 805 and 1909 (corresponding to the time 0 ns, 7.88 ns, 8.05 ns, and 19.09 ns) are marked with vertical lines. (TIF) [file pone.0084902.s005.tif]
