# Supplementary material for: Adaptation of an L-Proline Adenylation Domain to Use 4-Propyl-L-Proline in the Evolution of Lincosamide Biosynthesis
Source: PLoS One. 2013 Dec 27;8(12):e84902. doi: 10.1371/journal.pone.0084902 (PMC3874040; doi:10.1371/journal.pone.0084902)
Supplement: Table S1 — Overview of all tested combinations (A-domain vs. substrate) by biochemical assay. (PDF) [file pone.0084902.s006.pdf]

Table S1. Overview of all tested combinations (A-domain vs. substrate) by biochemical assay.

Protein	L-proline	EPL	PPL	BuPL	PePL	OH-L-proline	L-alanine	L-valine	L-tyrosine
CcbC	+ + +	-	-	NT	NT	+ +	+	+	-
LmbC	+	+ +	+ + +	+ + + +	+ + + +	+	+	+	-
LmbC G308V	+	+	+	NT	NT	NT	NT	NT	NT

EPL - (2*S*,4*R*)-4-ethyl-L-proline; PPL - (2*S*,4*R*)-4-propyl-L-proline; BuPL - (2*S*,4*R*)-4-butyl-L-proline; PePL - (2*S*,4*R*)-4-pentyl-L-proline and OH-L-proline - trans-4-hydroxy-L-proline. Adenylation activity is expressed by number of "+", when "+ + +" corresponds to the activity of the native A-domain with its natural substrate; "-" not detectable; NT - not tested.
