# Supplementary material for: Adaptation of an L-Proline Adenylation Domain to Use 4-Propyl-L-Proline in the Evolution of Lincosamide Biosynthesis
Source: PLoS One. 2013 Dec 27;8(12):e84902. doi: 10.1371/journal.pone.0084902 (PMC3874040; doi:10.1371/journal.pone.0084902)
Supplement: Protocol S1 — Preparation of (2S,4R)-4-alkyl-L-prolines. (PDF) [file pone.0084902.s007.pdf]

Preparation of (2*S*,4*R*)-4-alkyl-L-prolines

Chemistry

The synthesis of optically pure (2*S*,4*R*)-4-propyl-L-proline (**7**) started from protected intermediate **2**, which was prepared from commercially available L-pyroglutamic acid (**1**) by benzylation of its carboxylic group [1] followed by Boc-group introduction using Boc_2O/DMAP [2] (Scheme S1). The alkylation of the lithium enolate generated from **2** using LiHMDS by allyl bromide [3] led to a diastereoisomeric mixture of **3a** and **3b** in the ratio ca 1:2, which was separated by column chromatography (Scheme S1). A two-step, chemoselective reduction of the amide carbonyl group of **3b**, leading to protected propyl-L-proline **5** was accomplished as described previously [4]. Treatment of **5** with hydrogen on palladium led to the simultaneous hydrogenation of the double bond and benzyl group removal, yielding compound **6**, which gave, after acidic hydrolysis, propyl-L-proline hydrochloride (**7a**). This was finally neutralized by a methanolic solution of NH_3 to "free base" **7b**. The yield of *trans*-diastereoisomer **3b** was improved by inversion of the configuration at C-4 of **3a** using DBU [5].

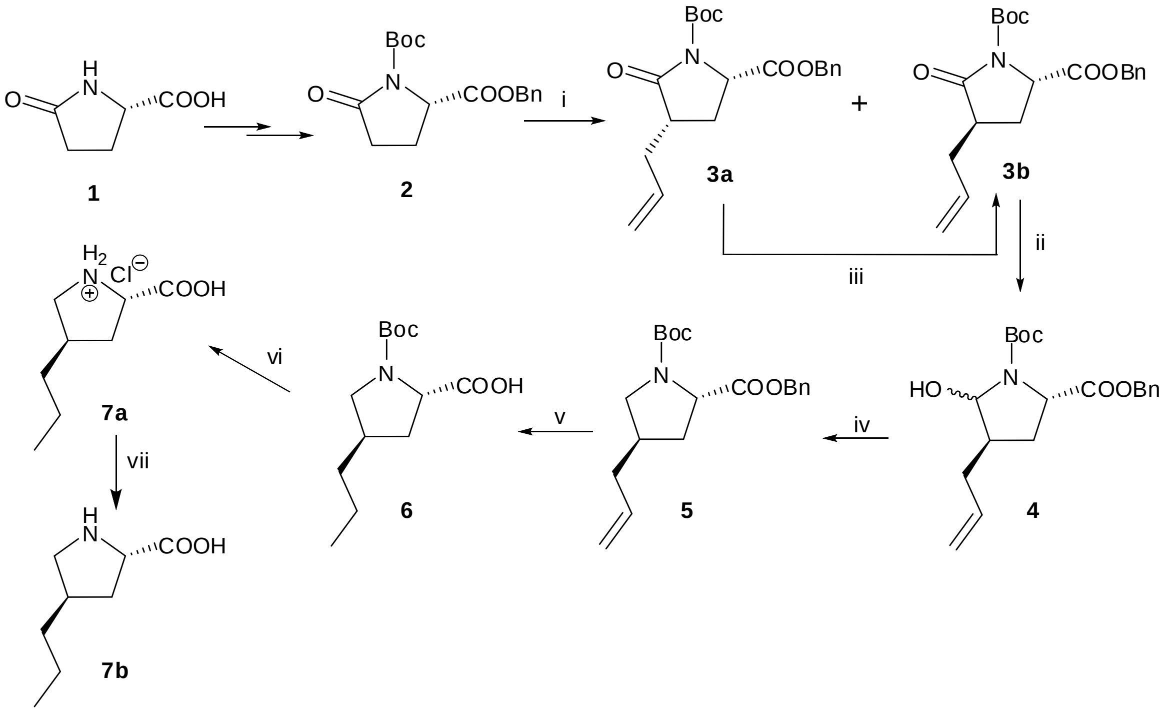

Scheme S1. Reagents and conditions. (i) a) LiHMDS, THF, –78°C, 1 h; b) allyl bromide (1.2 equiv.), –78°C, 75 min. (ii) a) $LiBHEt_3$, THF, –78°C, 0.5 h; b) H_2O_2, $NaHCO_3$, 0°C, 20 min.; (iii) DBU, CH_2Cl_2, 2 days, r.t.; (iv) $HSiEt_3$, $BF_3 \cdot Et_2O$, CH_2Cl_2, –78°C, 2 h; (v) H_2, Pd/C, EtOAc, 12 h, r.t.; (vi) HCl, EtOAc, 1 h; (vii) NH_3, MeOH, 10 min.

Preparation of (2*S*,4*R*)-4-ethyl-L-proline was based on the aldol condensation of protected pyroglutamate **8** with acetaldehyde (Scheme S2) [6]. The resultant diastereomeric mixture of the alcohols **9** was dehydrated to give predominately the *E*-isomers of the 4-alkylidenepyroglutamate **10** [6]. The mixture of both 4-alkylidenepyroglutamates **10** was hydrogenated using Pd on carbon, affording exclusively the *cis*-3,5-substituted 2-pyrrolidone **11a** [7]. The inversion of configuration at C-4 of **11a** was again achieved by the recently reported procedure [5] using DBU in CH_2Cl_2, which gave the key intermediate of the target compound – *trans*-3,5-substituted 2-pyrrolidone **11b**. A chemoselective, two-step reduction [8] of **11b** followed by acidic hydrolysis led to the final (2*S*,4*R*)-4-ethyl-L-proline (**13**).

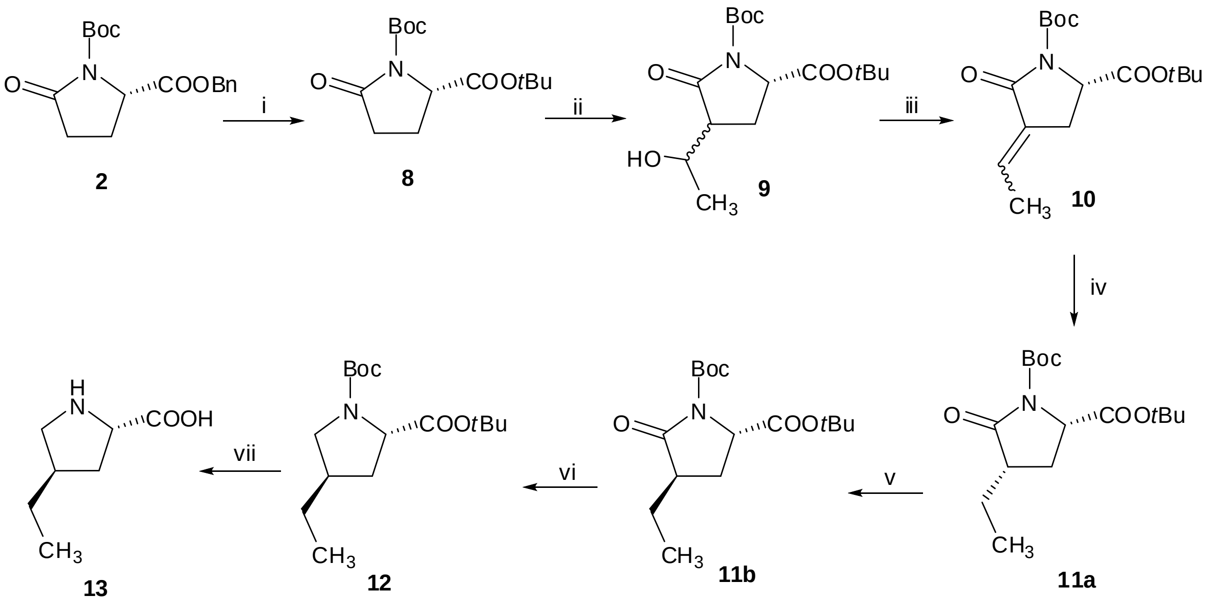

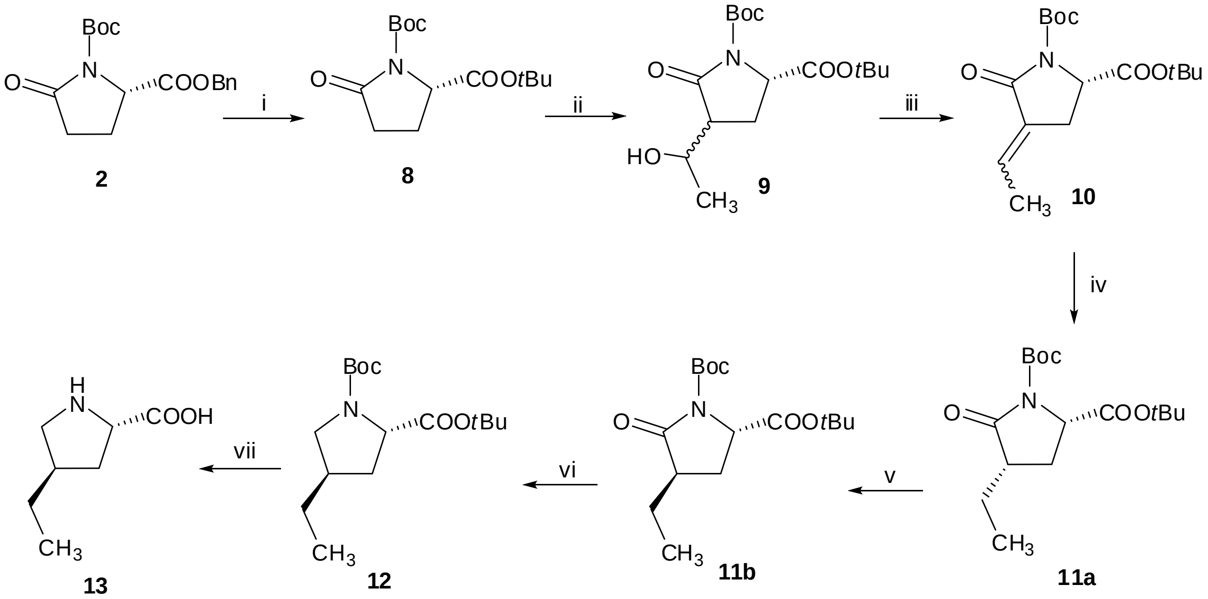

Scheme S2. Reagents and conditions. (i) a) H_2, Pd/C, EtOAc, 12 h, r.t.; b) *tert*-BuOH, DCC/DMAP, CH_2Cl_2, 12 h, r.t.; (ii) a) LiHMDS, THF, –78°C, 1 h; b) CH_3CHO (1.2 equiv.), $BF_3{\cdot}Et_2O$, –78°C, 75 min.; (iii) MsCl, Et_3N, CH_2Cl_2, 0.5 h at 0°C, then 2 days at r.t.; (iv) H_2, Pd/C, EtOAc, 48 h, r.t.; (v) DBU, CH_2Cl_2, 2 days, r.t.; (vi) a) $LiEt_3BH$, THF, –78°C, 0.5 h; b) H_2O_2, $NaHCO_3$, 0°C, 20 min.; c) Et_3SiH, $BF_3{\cdot}Et_2O$, CH_2Cl_2, –78°C, 2 h; (vii) a) HCl, 80°C, 2 h; b) NH_3, MeOH, 10 min.

Experimental

1. General methods

NMR spectra were recorded on a Bruker AVANCE III 400 MHz NMR (400.13 MHz for ^1H and 100.62 MHz for ^{13}C, Bruker BioSpin GmbH, Rheinstetten, Germany). Chemical shifts were referenced to the residual solvent signal of either $CDCl_3$ or DMSO-d_6 at 30°C (δ_H 7.265, δ_C 77.00; δ_H 2.50, δ_C 39.60). The digital resolution used justified reporting the proton and carbon chemical shifts to three and two decimal places, respectively. 2D NMR experiments (*g*COSY, ROESY, HOM2DJ, *g*HSQC; *g*HMBC) were performed using the manufacturer's software. Positive-ion electrospray ionization (ESI) mass spectra were recorded on a LCQ DECA spectrometer (ThermoQuest, San Jose, USA).

2. Chemicals

Solvents and reagents were purchased from Sigma-Aldrich and used without further purification. Compounds **2**, **3a**, **3b**, **4**, **5** and **8** were prepared as reported previously, [2,3,6] compound **8** was prepared from **2** [9]. Compound **11a** was prepared from **10** following a reported procedure [7].

(2*S*,4*R*)-4-Propyl-L-proline (7). Compound **5** (830 mg, 2.403 mmol) was dissolved in ethyl acetate (20 mL), Pd on carbon (110 mg; 10% w/w) was added and the resulting suspension was stirred under hydrogen for 12 h. The reaction mixture was filtered through Celite, the filtrate was evaporated and the crude carboxylic acid **6** was treated with 2 N HCl (1 mL) in EtOAc (30 mL) at 20°C for 1 h. The solvent was removed under reduced pressure and the crude solid was crystallized from a CH_2Cl_2/ethyl ether mixture to give **7a** as a colorless solid (290 mg, 62%).

Hydrochloride **7a** (290 mg, 1.497 mmol) was dissolved in MeOH (5 mL), treated with NH_3 (2 mL, 2 M solution in MeOH) and stirred for 10 min. The solvents were removed under reduced pressure and the evaporated residue was purified by chromatography on Avicel® PH-101 ($CH_3CN/H_2O/NH_4OH$ 100:15:1). Fractions containing pure **7b** were collected, evaporated, and the oily residue was precipitated by the addition of diethyl ether to give **7b** (201 mg, 86%) as a white amorphous solid.

^{1}H NMR (DMSO, 25°C): 0.814 (3 H, t, *J* = 7.1 Hz, 3 × H-3‘), 1.254 (2 H, m, 2 × H-2‘), 1.306 (2 H, m, 2 × H-1‘), 1.835 (1 H, ddd, *J* = 12.5, 9.5, 9.4 Hz, H-3u), 1.997 (1 H, m, H-4), 2.154 (1 H, ddd, *J* = 12.5, 6.8, 3.7 Hz, H-3d), 2.572 (1 H, dd, *J* =11.0, 9.9 Hz, H-5u), 3.365 (1 H, dd, *J* = 11.0, 7.0 Hz, H-5d), 3.785 (1 H, dd, *J* = 9.3, 3.6 Hz, H-2), 7.398 (2 H, s, NH + OH). ^{13}C NMR (DMSO, 25°C): 14.06 (C-3‘), 20.87 (C-2‘), 33.83 (C-1‘), 34.97 (C-3), 36.67 (C-4), 49.96 (C-5), 59.94 (C-2), 170.14 (C=O). MS (ESI): m/z 180.1 (M + Na^+).

***tert*-Butyl (2*S*)-L-(*tert*-butoxycarbonyl)-4-ethylidenepyroglutamate (10).** To a solution of *tert*-butyl *N*-BOC-pyroglutamate **8** (6 g, 21.028 15.6 mmol) in THF (50 mL) stirred at –78°C was added a 1 M solution of lithium hexamethyldisilazide in THF (25 mL, 25 mmol). The reaction mixture was stirred for 1 h at –78°C prior to the addition of a solution of the acetaldehyde (1.35 mL, 24.059 mmol) and $Et_2O{\cdot}BF_3$ (3 mL, 24.068 mmol) in THF (20 mL). The reaction mixture was stirred for 1 h at –78°C, quenched with saturated NH_4Cl solution (150 mL), and extracted with ethyl ether (3 × 75 mL). The combined organic phases were dried over Na_2SO_4, filtered, and evaporated to dryness. The crude mixture of aldols **9** was used without further purification in next reaction step.

The aldol mixture **9** was dissolved in CH_2Cl_2 (100 mL), cooled to 0°C and treated with methanesulfonyl chloride (2.68 mL, 34.4 mmol) and triethylamine (36 mL, 344 mmol). The resulting solution was stirred at 0°C for 30 min and then 2 days at room temperature, then it was evaporated to dryness, re-dissolved in a hexane/ethyl acetate mixture (100 mL, 1:1, v/v) and the precipitate formed was filtered off. The filtrate was evaporated and the reaction mixture was purified by flash chromatography (hexane/ethyl acetate 7:3) affording alkylidenepyroglutamate **10** (2.4 g, 36.7%) as a slightly yellow oil.

^{1}H NMR ($CDCl_3$, 30°C): 1.463 (9 H, s, O-*t*-Bu), 1.520 (9 H, s, N-Boc), 1.801 (3 H, ddd, *J* = 7.2, 2.2, 1.7, 3 × H-2‘), 2.545 (1 H, dddq, *J* = 17.3, 3.5, 2.6, 1.7, H-3u), 2.919 (1 H, dddq, *J* = 17.3, 10.2, 3.1, 2.2, H-3d), 4.489 (1 H, dd, *J* = 10.2, 3.5, H-2), 6.763 (1 H, ddq, *J* = 3.1, 2.6, 7.2). ^{13}C NMR ($CDCl_3$, 30 °C): 14.85 (C-2‘), 25.62 (C-3), 27.88 (O-*t*-Bu), 27.96 (N-Boc), 56.46 (C-2), 82.16 (C-O, *t*-Bu), 83.18 (C-O, N-Boc), 129.73 (C-4), 133.74 (C-1‘), 152.07 (1-CO), 165.92 (2-CO), 170.33 (2-CO). MS (ESI): m/z 334.2 (M + Na^+).

***tert*-Butyl (2*S*,4*R*)-L-(*tert*-butoxycarbonyl)-4-ethyl-pyroglutamate (11b).** The solution of pyroglutamate **11a** (2.2 g, 7.020 mmol) in CH_2Cl_2 (50 mL) was cooled to 0°C, DBU (3.75 mL, 25.076 mmol) was added and the resulting mixture was stirred for 1 h at 0°C and then for 48 h at room temperature. The reaction mixture was washed with a 0.1 M solution of HCl (2 × 50 mL), then with a saturated solution of $NaHCO_3$ (50 mL) and finally with water (50 mL). The organic layer was dried over anhydrous Na_2SO_4, evaporated and purified by flash chromatography (hexane/ethyl acetate 5:1) yielding **11b** (1.2 g, 55%) as a colorless oil.

^{1}H NMR ($CDCl_3$, 30°C): 0.955 (3 H, t, *J* = 7.5, 3 × H-2'), 1.418 (1 H, m, H-1'u), 1.477 (9 H, s, O-t-Bu), 1.507 (9 H, s, N-Boc), 1.922 (1 H, ddd, *J* = 13.2, 11.4, 9.7, H-3u), 1.924 (1 H, m, H-1'd), 2.117 (1 H, ddd, *J* = 13.2, 8.7, 1.6, H-3d), 2.527 (1 H, m, H-4), 4.423 (1 H, dd, *J* = 9.7, 1.6, H-2). ^{13}C NMR ($CDCl_3$, 30°C): 11.11 (C-2'), 23.43 (C-1'), 27.93 (2 × *t*-Bu), 28.08 (C-3), 42.93 (C-4), 57.78 (C-2), 82.18 (C-O, O-*t*-Bu), 83.11 (C-O, N-Boc), 149.56 (1-CO), 170.42 (2-CO), 175.07 (C-5). MS (ESI): m/z 336.2 (M + Na^+).

(2*S*,4*R*)-4-Ethyl-L-proline (13). 1.0 M solution of lithium triethylborohydride in THF (2.22 mL, 2.22 mmol) was added to a solution of **11b** (580 mg, 1.851 mmol) in THF (8 mL) at –78°C under argon and stirred for 30 min. The reaction was quenched with saturated aqueous $NaHCO_3$ (2.5 mL) and warmed to 0°C. H_2O_2 (7 drops, 30% w/w) was added, and the mixture was stirred at 0°C for 20 min. The organic solvent was then removed *in vacuo,* and the aqueous layer was extracted with CH_2Cl_2 (3 × 10 mL). The combined organic layers were dried over Na_2SO_4, filtered, and concentrated. The crude reaction mixture was used without further purification and dissolved in CH_2Cl_2 (20 mL). After the addition of triethylsilane (0.29 mL, 1.864 mmol) the mixture was cooled to –78°C. Boron trifluoride etherate (0.248 mL, 2.039 mmol) was added dropwise under argon. A second portion of triethylsilane (0.29 mL) and boron trifluoride etherate (0.248 mL) were added after 30 min, allowing the reaction to reach room temperature. The reaction mixture was stirred at room temperature for 30 min, then was the reaction quenched with saturated aqueous $NaHCO_3$ (5 mL), extracted with CH_2Cl_2 (3 × 10 mL), and dried over Na_2SO_4. Evaporation of the solvent and purification by flash chromatography (hexane/ethyl acetate 4:1) yielded L-proline *tert*-butyl ester which was hydrolyzed for 2 h at 85°C with 6 N HC1 (20 mL). The resulting solution was evaporated to dryness, and the solid was crystallized from CH_2Cl_2/diethyl ether affording pure **13** (192 mg, 58%). The hydrochloride of **13** (192 mg, 1.068 mmol) was neutralized to "free base" **13** (142 mg, 93%) by the same procedure described for **7**.

^{1}H NMR (DMSO, rt): 0.834 (3 H, t, *J* = 7.4 Hz, 3 × H-2'), 1.323 (2 H, m, 2 × H-1'), 1.707 (1 H, ddd, *J* = 12.6, 6.9, 3.9 Hz, H-3u), 1.939 (1 H, m, H-4), 2.126 (1 H, ddd, *J* = 12.6, 9.3, 9.3 Hz, H-3d), 2.642 (1 H, dd, *J* =11.3, 9.5 Hz, H-5u), 3.369 (1 H, dd, *J* = 11.3, 7.1 Hz, H-5d), 3.800 (1 H, hidden under water, H-2), 7.398 (2 H, s, NH + OH). ^{13}C NMR (DMSO, rt): 12.79 (C-2'), 25.00 (C-1'), 34.91 (C-3), 39.04 (C-4), 50.31 (C-5), 60.61 (C-2), 171.12 (C=O). MS (ESI): m/z 166.1 (M + Na^+).

References:

[1] Gosselin F, Lubell WD (2000) Rigid dipeptide surrogates: syntheses of enantiopure quinolizidinone and pyrroloazepinone amino acids from a common diaminodicarboxylate precursor. J Org Chem 65: 2163-2171.

[2] Gloanec P, Herve Y, Bremand N, Lecouve JP, Breard F, De Nanteuil G (2002) Synthesis of benzyl (6S)-1,3-dichloro-4-oxo-4,6,7,8-tetrahydro-pyrrolo[1,2-a]pyrazine-6-carboxylic ester, a new conformationally constrained peptidomimetic derivative. Tetrahedron Lett 43: 3499-3501.

[3] Llinàs-Brunet M, Bailey M, Fazal G, Ghiro E, Gorys V, Goulet S, Halmos T, Maurice R, Poirier M, Poupart MA, Rancourt J, Thibeault D, Wernic D, Lamarre D (2000)

Highly potent and selective peptide-based inhibitors of the hepatitis C virus serine protease: towards smaller inhibitors. Bioorg Med Chem Lett 10: 2267-2270.

[4] Ezquerra J, Pedregal C, Rubio A, Yruretagoyena B, Escribano A, Sanchez-ferrando F (1993) Stereoselective reactions of lithium enolates derived from N-BOC protected pyroglutamic esters Tetrahedron 49: 8665-8678.

[5] Tarver JE, Terranova KM, Joullie MA (2004) Hetero-Diels-Alder and pyroglutamate approaches to (2*S*,4*R*)-2-methylamino-5-hydroxy-4-methylpentanoic acid. Tetrahedron 60: 10277-10284.

[6] Ezquerra J, Pedregal C, Yruretagoyena B, Rubio A, Carreno MC, Escribano A, Ruano JLG (1995) Synthesis of enantiomerically pure 4-substituted glutamic acids and prolines - general aldol reaction of pyroglutamate lactam lithium enolate mediated by Et(2)O-Center-Dot-BF3. J Org Chem 60: 2925-2930.

[7] Moody CM, Young DW (1994) Stereospecific synthesis of 4-alkylglutamates and 4-alkylprolines. Tetrahedron Lett 35: 7277-7280.

[8] Pedregal C, Ezquerra J, Escribano A, Carreno MC, Ruano JLG (1994) Highly chemoselective reduction of N-Boc protected lactams. Tetrahedron Lett 35: 2053-2056.

[9] Baldwin JE, Miranda T, Moloney M, Hokelek T (1989) Amino-acid synthesis using (L)-pyroglutamic acid as a chiral starting material. Tetrahedron 45: 7459-7468.
